# Selectivity of Nitrate Reduction and Hydrogen Evolution Reaction Modulated by Aggregation of Carbon-Supported Fe Species

**DOI:** 10.1021/acs.langmuir.6c03729

**Published:** 2026-09-03

**Authors:** Wun-Yan Wu, Cheng-Jhe Tsai, Chih-Ying Lin, Serhii Makovetskyi, Ting-Shan Chan, Yao-Chang Lee, Ying-Rui Lu, Wen-Feng Liaw

**Affiliations:** † Department of Chemistry, 34881 National Tsing Hua University, Hsinchu 30013, Taiwan; ‡ 57815 National Synchrotron Radiation Research Center, Hsinchu 300092, Taiwan

## Abstract

Fe-species distribution plays an important role in electrochemical nitrate reduction, yet its influence on the competing hydrogen evolution reaction (HER) remains insufficiently understood. Herein, carbon-supported Fe catalysts with distinct Fe-species distributions were prepared by controlling the Fe precursor concentration. **FeAC** consists of highly dispersed atomic-scale Fe species and FeO*_x_* nanoclusters, whereas **Fe**_**2**_ **O**_**3**_ **/FeAC** additionally contains crystalline α-Fe_2_O_3_ nanoparticles. X-ray absorption spectroscopy, X-ray diffraction, and electron microscopy confirm the distinct Fe dispersion states in the two catalysts. Electrochemical measurements reveal that **FeAC** achieves significantly higher NH_3_ Faradaic efficiency (79.6 ± 2.2%) than **Fe**_**2**_ **O**_**3**_ **/FeAC** (37.8 ± 3.1%) at −0.8 V versus RHE, whereas **Fe**_**2**_ **O**_**3**_ **/FeAC** exhibits a higher NH_3_ production rate accompanied by enhanced HER. Comparable electrochemically accessible surface areas and interfacial charge-transfer characteristics indicate that the observed catalytic differences are not primarily governed by electrochemical surface area or charge-transfer properties. *Operando* Raman spectroscopy reveals distinct surface reaction environments, while operando ATR-SEIRAS together with EPR measurements indicates enhanced formation of hydrogen-related Fe–H intermediates and hydrogen radicals on **Fe**_**2**_ **O**_**3**_ **/FeAC**, consistent with its stronger HER activity. In contrast, the absence of detectable Fe–H species on **FeAC** is associated with suppressed hydrogen adsorption and improved nitrate reduction selectivity. These results demonstrate that controlling Fe-species distribution provides an effective strategy for balancing activity and selectivity in carbon-supported Fe electrocatalysts for electrochemical nitrate reduction.

## Graphical abstract

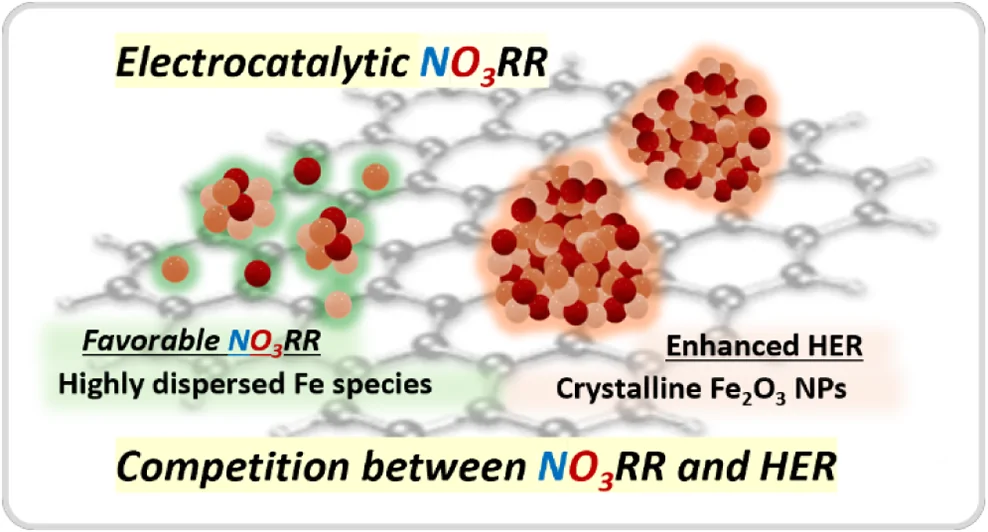

## Introduction

Nitrate contamination has become an increasingly important environmental issue due to the excessive use of nitrogen-containing fertilizers, industrial emissions, and wastewater discharge.^1,2^ The accumulation of nitrate in groundwater and surface water not only leads to eutrophication but also causes potential risks to human health.^1^ Therefore, the development of efficient nitrate removal and conversion technologies has attracted considerable attention. Among various approaches, the electrocatalytic nitrate reduction reaction (NO_3_RR) is considered to be one of the most promising strategies because it can simultaneously achieve environmental remediation and value-added chemical production under mild conditions.^1,3–6^ Nitrate can be converted into ammonia (NH_3_), which is an essential chemical feedstock for fertilizers, pharmaceuticals, and emerging energy applications. Compared with the conventional Haber–Bosch process, which requires high temperature, high pressure, and intensive fossil fuel consumption, electrocatalytic nitrate-to-ammonia conversion provides a more sustainable route powered by renewable electricity.^4,5,7–10^ However, the NO_3_RR process involves multiple proton-coupled electron transfer steps and strongly competes with the hydrogen evolution reaction (HER), resulting in limited NH_3_ selectivity and Faradaic efficiency. Therefore, the design of catalysts capable of stabilizing nitrate reduction intermediates while suppressing HER is critical for improving catalytic performance. Recent studies have shown that surface-conjugated molecular catalysts, highly dispersed metal species, nanoclusters, nanoparticles, and bimetallic alloys/heterostructures can effectively regulate the adsorption behavior of nitrate-derived intermediates and suppress undesired HER activity.^3,11–21^

Iron-based catalysts are particularly attractive because of their low cost, earth abundance, and favorable interaction with nitrogen-containing intermediates.^8^ Fe-based nanoparticle-carbon hybrid electrocatalysts can achieve highly efficient electrochemical NO_3_RR.^22^ Recently, the γ-Fe_2_O_3_/BCN catalyst exhibited a high ammonia production rate and excellent cycling stability, indicating effective suppression of the competing HER. Interfacial charge transfer between γ-Fe_2_O_3_ and BCN, modulating the electronic structure and enhancing NO_3_RR activity and NH_3_ selectivity, was revealed by DFT calculations.^23^ The porous carbon discs decorated with α-Fe/Fe_3_O_4_ nanoparticles exhibit high NH_3_ yield rates together with energy efficiencies, indicating that the α-Fe/Fe_3_O_4_ heterostructure plays complementary catalytic roles during NO_3_RR, where α-Fe facilitates nitrate deoxygenation while Fe_3_O_4_ promotes the subsequent hydrogenation steps toward NH_3_ formation.^24^

Iron- and nitrogen-coordinated carbon (Fe–N–C) has emerged as an effective platform for stabilizing atomically dispersed Fe sites. Atomically dispersed Fe single-atom catalysts (SAC) exhibit high activity and selectivity toward electrochemical NO_3_^–^-to-NH_3_. The isolated Fe sites were proposed to influence the adsorption behavior of nitrogen-containing intermediates and suppress the N–N coupling pathway for N_2_ formation due to the absence of adjacent metal centers, thereby enhancing NH_3_ selectivity.^15,16,25^ Recently, an electrified nanoporous membrane integrated with Fe SACs enhanced nitrate reduction through confined mass transport and intensified local reactant enrichment. The nanoporous architecture significantly improved the atomic utilization efficiency of Fe active sites, enabling highly selective and efficient NO_3_^–^-to-NH_3_ conversion under continuous-flow conditions.^17^

The catalytic behaviors of highly dispersed Fe species (SAC, DAC, TAC, and FeO*_x_* nanoclusters) and Fe-based nanoparticles show significant differences, especially in terms of NH_3_ selectivity and HER competition. Therefore, understanding the structure–activity relationship between Fe active sites and nitrate reduction performance is essential for the rational design of efficient electrocatalysts for ammonia production.^24,26–28^ A synergistic catalytic system consisting of atomically dispersed Fe sites and ultrasmall Fe clusters was constructed on nitrogen-doped hollow carbon tubes through a facile low-temperature calcination strategy coupled with confined surface crystallization. The coexistence of an isolated Fe SAC and Fe nanoclusters enabled cooperative nitrate adsorption and intermediate conversion, thereby promoting efficient electrochemical nitrate reduction to yield ammonia.^24,28,29^

Recent studies have further suggested that integrating atomically dispersed Fe sites with iron oxide nanoparticles can create synergistic catalytic interfaces rather than acting as isolated active phases.^30^ Specifically, α-/γ-Fe_2_O_3_ nanoparticles supported on Fe–N–C have been shown to deliver industrially relevant NH_3_ partial current densities while maintaining high selectivity, highlighting the potential advantages of integrating isolated Fe sites and iron oxide nanoparticles within a single catalyst architecture. Nevertheless, direct comparisons between highly dispersed Fe species and Fe_2_O_3_ nanoparticle-containing catalysts prepared on the same carbon support remain limited. Consequently, the influence of Fe-species distribution on the balance between nitrate reduction and the competing HER has not been clearly established. Herein, sulfur-doped carbon-supported Fe catalysts with distinct Fe-species distributions were prepared by controlling the Fe precursor concentration. **FeAC** consists of atomically dispersed Fe species mixed with FeO*_x_* nanoclusters, while **Fe**_**2**_ **O**_**3**_ **/FeAC** additionally contains dispersed α-Fe_2_O_3_ nanoparticles. Structural characterization was combined with electrochemical measurements and *operando* spectroscopic analyses to correlate Fe-species distribution with nitrate reduction selectivity and HER activity.

## Results and Discussion

### Material Synthesis and Characterization of **FeAC** and **Fe_2_O_3_/FeAC**

Metal-coordinated nitrogen-containing polymer precursors have been widely adopted in electrochemical catalysis due to their ability to improve metal dispersion and suppress severe aggregation during thermal treatment.^31–33^ Among them, a nitrogen-rich coordination matrix via pyrolysis of poly­(*o*-phenylenediamine) (PoPDA) facilitates homogeneous metal-ion dispersion and promotes the formation of M–Nx active sites, nanoclusters, and nanoparticles within conductive and structurally stable carbon frameworks.^31,34–36^ Synthetic procedures for the iron/carbon-based composite materials **FeAC** and **Fe**_**2**_ **O**_**3**_ **/FeAC** are illustrated in Scheme 1. The materials were prepared via precoordination of Fe^3+^ with PoPDA by immersing equal amounts of PoPDA in 0.02 and 0.10 mM FeCl_3_ solutions, with all other synthetic conditions kept identical. Specifically, a mixture of thiourea-treated active carbon black (**SCB**) and the various ratio of Fe:PoPDA precursors were transferred into a tubular furnace then pyrolysis at 700 °C with a heating rate of 0.1 °C min^–1^ under nitrogen for 4 h. This procedure was employed to introduce the multi-iron-chelated PoPDA precursor into **SCB**, thereby enabling defect-site functionalization. The annealing process induced the incorporation of highly dispersed Fe species into the carbon-based template at lower Fe precursor concentrations, leading to the formation of atomically dispersed Fe mixed with FeO*_x_* nanoclusters composite material **FeAC**. In contrast, the annealing of higher Fe precursor concentrations promoted the aggregation of Fe species on the carbon support, resulting in the coexistence of larger size iron oxide nanoparticles and **FeAC** composite material **Fe**_**2**_ **O**_**3**_ **/FeAC**.

**Scheme 1:**
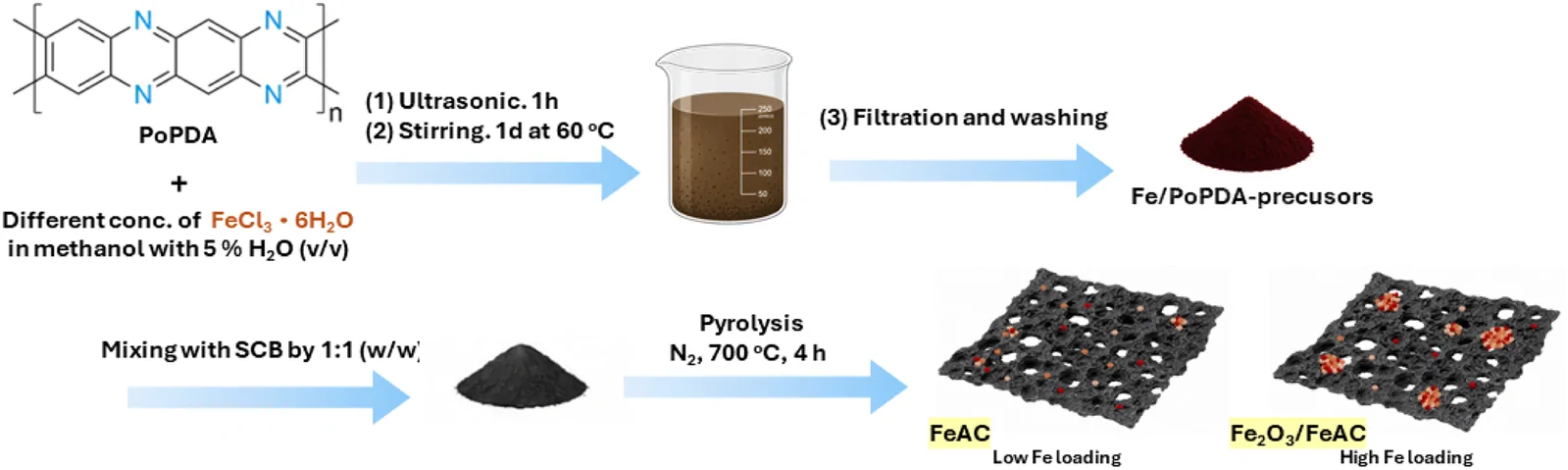
Scheme for the Preparation of **FeAC** and **Fe**_**2**_ **O**_**3**_/FeAC[Fn sch1-fn1] [Fn sch1-fn2]

a
SCB = thiourea-treated active carbon black.

b
As-prepared sample information present in Table S1.

ICP-MS analysis confirmed that the Fe contents of **FeAC** and **Fe**_**2**_ **O**_**3**_ **/FeAC** were 0.88 ± 0.05 wt % and 1.52 ± 0.04 wt %, respectively (Table S2). The higher Fe loading in **Fe**_**2**_ **O**_**3**_ **/FeAC** is consistent with the higher precursor concentration used during synthesis and the formation of Fe_2_O_3_ nanoparticles, as observed by TEM and XRD. Elemental analysis revealed sulfur and nitrogen contents of 3.02% and 8.24% for **FeAC**, and 1.92% and 4.89% for **Fe**_**2**_ **O**_**3**_ **/FeAC**, respectively. Powder X-ray diffraction patterns of **FeAC** and **Fe**_**2**_ **O**_**3**_ **/FeAC** showed two broad diffraction peaks at 23.2° and 43.6°, corresponding to the carbon black support (Figure S1). In addition, **Fe**_**2**_ **O**_**3**_ **/FeAC** exhibited diffraction peaks at 24.2°, 33.2°, 35.6°, 43.7°, 49.5°, 54.1°, 57.6°, 62.5°, 64.0°, and 72.0°, which can be indexed to the *R*3̅*c* α-Fe_2_O_3_ phase dispersed on the carbon-based template (Figure S1). The FTIR spectra of **FeAC** and **Fe**_**2**_ **O**_**3**_ **/FeAC** (Figure S1) displaying peaks at 1630–1500 cm^–1^and 1400–1000 cm^–1^ are attributed to in-plane C=N/C=C/C=O vibration and C–N/C–C/C–O vibrational bands, respectively, originating from the pyrolysis of PoPDA with SCB substrate.

The morphologies of **FeAC** and **Fe**_**2**_ **O**_**3**_ **/FeAC** were analyzed using field-emission scanning electron microscopy (FESEM). The **FeAC** sample showed no significant particles on the carbon-based substrate, while **Fe**_**2**_ **O**_**3**_ **/FeAC** exhibited roughly spherical iron oxide particles dispersed on the carbon-based substrate with particle sizes ranging from approximately 60 to 120 nm (Figures S2 and S3). Backscattered electron (BSE) SEM images of the **FeAC** sample showed no detectable iron-based particle dispersion. Energy-dispersive X-ray spectroscopy (EDX) mapping (Figures S4 and S5) of **FeAC** and **Fe**_**2**_ **O**_**3**_ **/FeAC** indicated that **FeAC** was primarily composed of C (86.8%), N (8.3%), O (3.6%), S (1.2%), and undetectable Fe, while **Fe**_**2**_ **O**_**3**_ **/FeAC** consisted of C (83.5%), N (5.6%), O (8.4%), S (1.0%), and Fe (1.6%). The distributions of C and N were relatively homogeneous across the surface, while localized Fe and O signals in **Fe**_**2**_ **O**_**3**_ **/FeAC** suggested the formation of surface-dispersed iron oxide particles. Low-magnification spherical-aberration-corrected field-emission TEM (AC-TEM) images of **FeAC** revealed the aggregated carbonaceous nanosheets without observable crystalline Fe-containing nanoparticles (Figures 1 and S6). In contrast, **Fe**_**2**_ **O**_**3**_ **/FeAC** exhibited distinct iron oxide nanoparticles anchored on the carbon support, as evidenced by the clear lattice fringes observed in the bright-field AC-TEM image. The measured interplanar spacings of 0.25 nm can be assigned to the (110) plane of crystalline α-Fe_2_O_3_. In addition, the corresponding SAED pattern further confirmed the presence of crystalline α-Fe_2_O_3_ domains in the **Fe**_**2**_ **O**_**3**_ **/FeAC** catalyst.^37,38^ High-magnification bright-field TEM images of **FeAC** further showed only disordered carbon domains without obvious lattice fringes attributable to crystalline iron oxide particles. In the corresponding HAADF-STEM images, numerous isolated bright spots were observed in selected regions of the carbon matrix for both **FeAC** and **Fe**_**2**_ **O**_**3**_ **/FeAC** owing to the large atomic-number contrast between Fe species and the carbon-based support. For **FeAC**, these bright features are attributed to atomically dispersed Fe mixed with FeO*_x_* nanoclusters distributed throughout the carbon matrix. For **Fe**_**2**_ **O**_**3**_ **/FeAC**, the larger bright domains corresponding to iron oxide nanoparticles were also observed, indicating the coexistence of isolated atomic-scale Fe, FeO*_x_* nanoclusters, and Fe_2_O_3_ nanoparticles on the carbon support (Figures 1 and S6).

**Figure 1:**
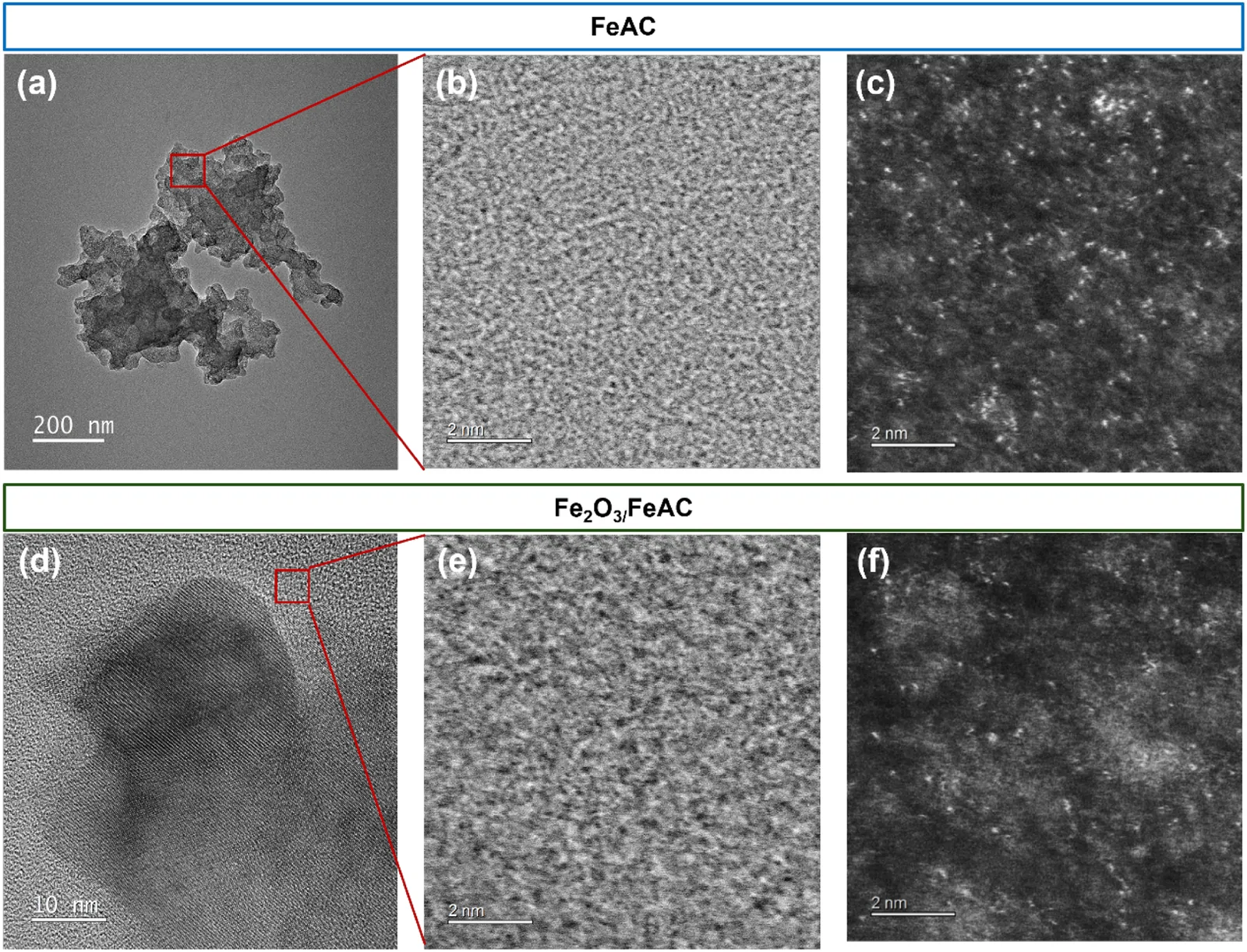
(a) Low-magnification AC-TEM image of **FeAC**. (b) High-magnification bright-field TEM image of **FeAC**, revealing a disordered carbon structure without observable Fe nanoparticles. (c) High-magnification HAADF-STEM image of **FeAC**, showing atomically dispersed Fe species distributed throughout the carbon matrix. (d) Low-magnification AC-TEM image of **Fe_2_O_3_/FeAC**, showing crystalline Fe_2_O_3_ nanoparticles dispersed on the carbon support. (e) Enlarged bright-field TEM image of the carbon matrix in **Fe_2_O_3_/FeAC**. (f) High-resolution HAADF-STEM image showing atomically dispersed Fe species, with representative atomic sites highlighted by red circles. The **Fe_2_O_3_/FeAC** specimen was prepared by focused ion beam (FIB) sectioning to obtain electron-transparent cross-sectional regions for high-resolution TEM analysis.

X-ray photoelectron spectroscopy (XPS) was employed to investigate the surface chemical states and elemental environments of **FeAC** and **Fe**_**2**_ **O**_**3**_ **/FeAC**, particularly for Fe, O, N, and S species associated with the carbon-supported Fe active sites and iron oxide phases (Figure 2). Prior to peak fitting, a Shirley background was subtracted from all spectra. Peak fitting was subsequently performed using mixed Gaussian–Lorentzian line shapes following commonly accepted procedures. For the Fe 2p spectra, both **FeAC** and **Fe**_**2**_ **O**_**3**_ **/FeAC** exhibited characteristic Fe 2p_3/2_ and Fe 2p_1/2_ peaks at 710.5 and 723.9 eV, respectively, together with the corresponding satellite peaks, suggesting the presence of Fe^3+^-dominated species. Minor Fe^2+^ features at 708.9 and 721.3 eV were also observed, which could be partially attributed to photoreduction effects during X-ray irradiation (Figure 2a and e).^39–41^ The O 1s XPS spectra of **FeAC** and **Fe**_**2**_ **O**_**3**_ **/FeAC** were deconvoluted into three main components located at approximately 529.9, 531.3, and 532.5 eV, which can be assigned to lattice Fe–O, hydroxyl or defect oxygen species, C–O/C=O functionalities from the carbon matrix.^39,42^ The peak at 529.9 eV is generally associated with lattice oxygen in Fe–O coordination environments, suggesting the presence of iron oxide species on the carbon support. Compared to **FeAC**, **Fe**_**2**_ **O**_**3**_ **/FeAC** shows a relatively stronger contribution from lattice Fe–O and a slightly lower proportion of C–O/C=O species, which may indicate partial conversion of surface oxygen-containing functional groups into Fe–O coordination environments during iron oxide formation. In addition, the signal at 531.3 eV is commonly attributed to hydroxyl groups or defect-related oxygen species and may also reflect the presence of low-coordination Fe–O sites at the interface (Figure 2b and f).^43–45^ The obtained results support the coexistence of isolated atomic-scale Fe and FeO*_x_* nanoclusters on the carbon matrix.^46,47^ For the N 1s spectra, **FeAC** and **Fe**_**2**_ **O**_**3**_ **/FeAC** show similar features and can be deconvoluted into pyridinic-N (398.5 eV), pyrrolic-N and/or Fe–Nx species (399.9 eV), and graphitic-N (401.5 eV) (Figure 2c and g).^15,48–50^ This indicates that nitrogen-containing functionalities remain incorporated within the carbon matrix and may contribute to the stabilization of dispersed Fe species. The relatively higher nitrogen content in **FeAC** further suggests a greater abundance of N-containing sites capable of interacting with highly dispersed Fe species through Fe–N coordination. The S 2p spectra of **FeAC** and **Fe**_**2**_ **O**_**3**_ **/FeAC** exhibit two peaks at 163.5 and 164.7 eV, corresponding to S 2p_3/2_ and S 2p_1/2_, respectively, which can be assigned to thioether-like sulfur species within the carbon framework (Figure 2d and h).^51–53^ That is, sulfur-containing functionalities indicate sulfur incorporation into the carbon matrix and may contribute to the modulation of the local electronic structure of the support.

**Figure 2:**
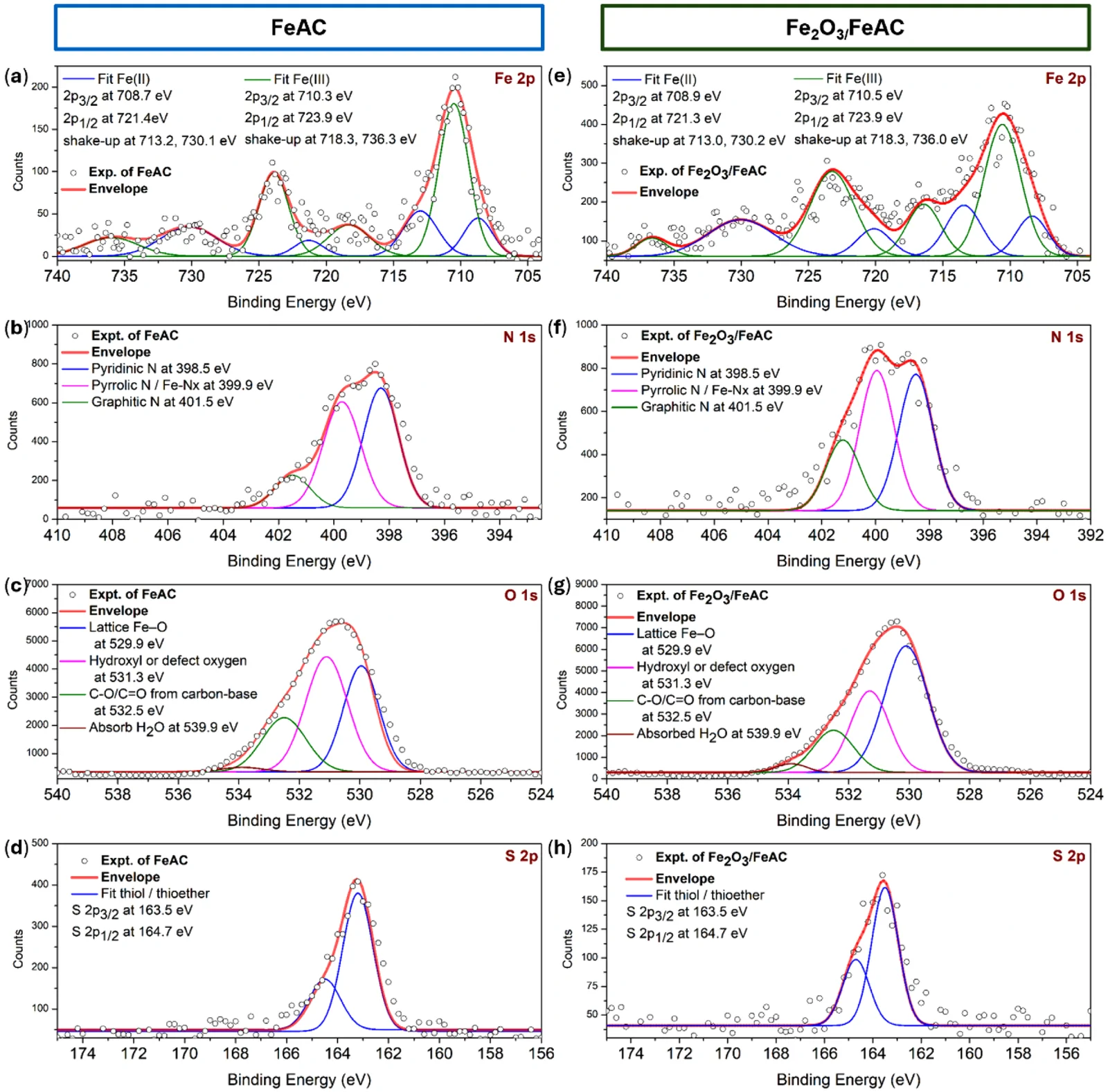
High-resolution XPS spectra of **FeAC** and **Fe_2_O_3_/FeAC**. (a, e) Fe 2p, (b, f) N 1s, (c, g) O 1s, and (d, h) S 2p spectra of **Fe_2_O_3_/FeAC** and **FeAC**, respectively.

X-ray absorption spectroscopy (XAS) at the Fe K-edge was further employed to investigate the local coordination environment and oxidation state of Fe in **FeAC** and **Fe**_**2**_ **O**_**3**_ **/FeAC** (Figure 3). The normalized Fe K-edge XANES spectra of **FeAC** and **Fe**_**2**_ **O**_**3**_ **/FeAC** are both similar to that of the Fe_2_O_3_ standard, indicating that the Fe in both materials are Fe^3+^ dominant. Although **FeAC** and **Fe**_**2**_ **O**_**3**_ **/FeAC** show similar inflection points at 7112.9 eV close to that of the Fe_2_O_3_ reference, **FeAC** exhibits a stronger white-line intensity and a slightly higher-energy main derivative peak, suggesting a different local Fe coordination environment rather than a change in Fe oxidation state. This difference may be associated with the coexistence of isolated atomic-scale Fe and nanoclusters in **FeAC** (Figure 3a–b). The Fourier-transformed *k* ^3^-weighted EXAFS spectrum of **FeAC** shows a dominant first-shell feature in the *R*-space region of approximately 1.4–1.8 Å. EXAFS fitting assigns this contribution to Fe–O/N coordination with a coordination number of 3.13 and a bond distance of 1.78 Å, together with two Fe···Fe contributions with a coordination number of 1.32 at 2.48 Å and 1.17 at 2.97 Å, respectively. (Figure 3c–d). The EXAFS results of **FeAC** reveal low-coordination Fe–O/N environments with minor Fe···Fe contributions, while the absence of long-range ordering in XRD and the presence of isolated bright features in HAADF-STEM are consistent with coexistence of isolated atomic-scale Fe and FeO*_x_* nanoclusters instead of well-defined crystalline nanoparticles. In comparison, the Fourier-transformed *k* ^3^-weighted EXAFS spectrum of **Fe**_**2**_ **O**_**3**_ **/FeAC** exhibits a similar first-shell feature in the *R*-space region of approximately 1.4–2.0 Å. EXAFS fitting gives an Fe–O/N coordination number of 3.93 with a bond distance of 1.80 Å. In addition, Fe–Fe scattering paths are observed at 2.45 and 2.94 Å, with coordination numbers of 0.21 and 2.00, respectively, indicating the presence of more ordered Fe···Fe interactions (Figure 3e–f). These features are consistent with the formation of crystalline iron oxide nanoparticles in addition to dispersed Fe species on the carbon substrate. Overall, the XAS results, together with XRD and electron microscopy analyses, support the conclusion that **FeAC** is composed of highly dispersed atomic-scale Fe and FeO*_x_* nanoclusters, while **Fe**_**2**_ **O**_**3**_ **/FeAC** consists of dispersed atomic-scale Fe, FeO*_x_* nanoclusters, and crystalline iron oxide nanoparticles.

**Figure 3:**
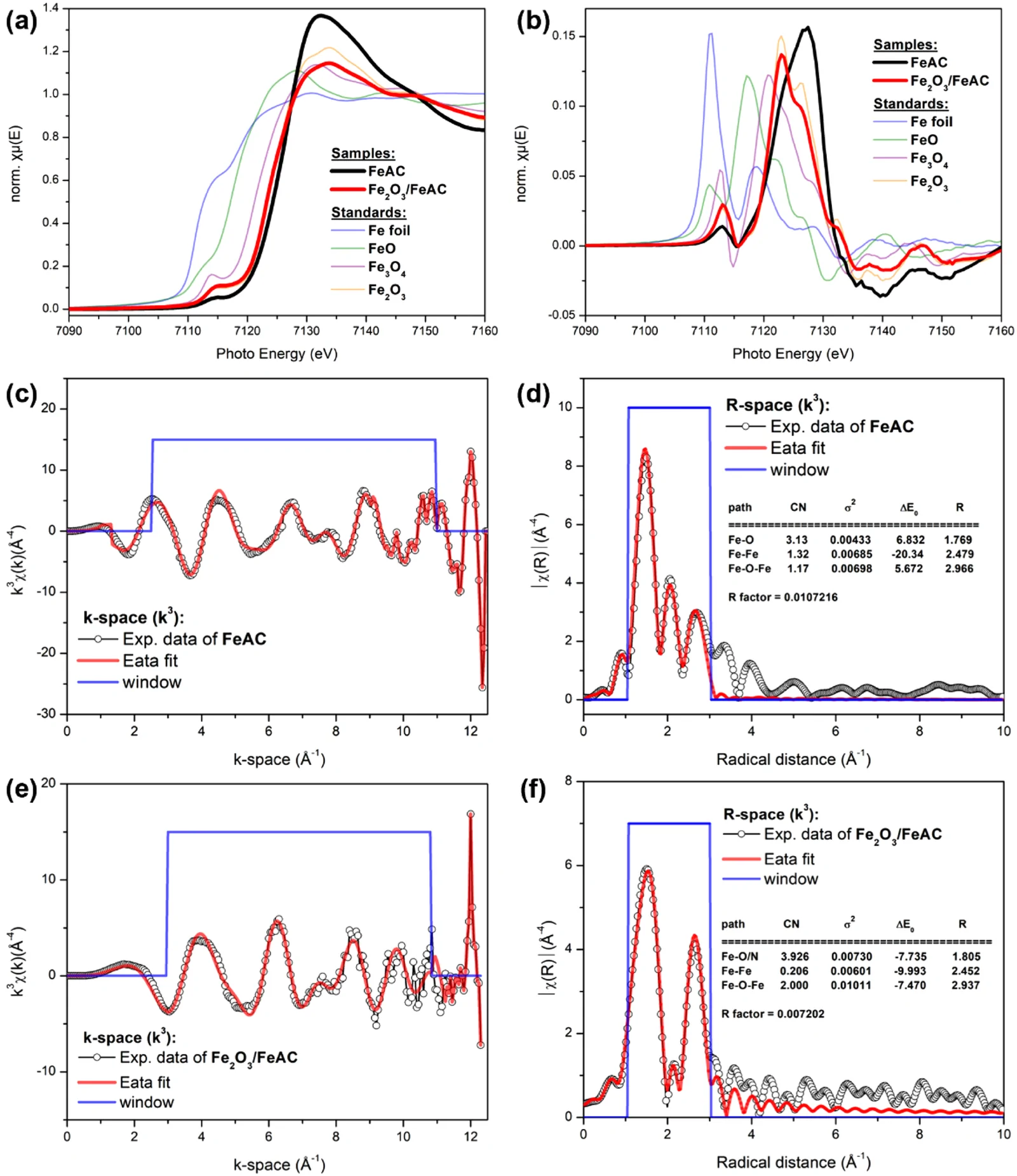
XAS analysis of **FeAC** and **Fe_2_O_3_/FeAC**. (a) Normalized Fe K-edge XANES spectra of **FeAC** and **Fe_2_O_3_/FeAC**, together with Fe foil, FeO, Fe_3_O_4_, and Fe_2_O_3_ reference compounds. (b) First-derivative Fe K-edge XANES spectra, showing that the average Fe oxidation state of both samples is close to Fe^3+^. (c) *k*-space EXAFS fitting of **FeAC** and (d) *R*-space EXAFS fitting of **FeAC**. (e) *k*-space EXAFS fitting of **Fe_2_O_3_/FeAC** and (f) *R*-space EXAFS fitting of **Fe_2_O_3_/FeAC**.

### Electrocatalytic Performance of Nitrate Reduction Reaction

Fe/C-based materials (**FeAC** and **Fe**_**2**_ **O**_**3**_ **/FeAC**) and **SCB** without Fe loading were prepared for electrochemical measurements by dispersing 1 mg of catalyst in a Nafion-containing suspension, followed by sonication and drop-casting onto carbon fiber paper (**CFP**). Linear sweep voltammetry (LSV) measurements were carried out in 0.1 M NaNO_3_ and 0.1 M Na_2_SO_4_ electrolyte to evaluate the nitrate reduction performance of **SCB/CFP**, **Fe**_**2**_ **O**_**3**_ **/FeAC/CFP**, and **FeAC/CFP**. As shown in Figure 4, both **Fe**_**2**_ **O**_**3**_ **/FeAC/CFP** and **FeAC/CFP** exhibit substantially enhanced cathodic current densities compared to **SCB/CFP**, suggesting that the introduction of Fe species facilitates nitrate reduction under cathodic polarization. Among the Fe-containing catalysts, **Fe**_**2**_ **O**_**3**_ **/FeAC/CFP** shows the highest overall current density, which may be associated with the larger amount of Fe-based active sites and the presence of Fe_2_O_3_ nanoparticles. The NH_3_ yield rates at an applied potential of −0.8 V vs RHE are summarized in Figure 4. **Fe**_**2**_ **O**_**3**_ **/FeAC/CFP** exhibits the highest NH_3_ yield rate of 161.4 μmol·h^–1^·cm^–2^, followed by **FeAC/CFP** with 133.1 μmol·h^–1^·cm^–2^, while **SCB/CFP** only gives 28.3 μmol·h^–1^·cm^–2^. The higher NH_3_ yield rate observed for **Fe**_**2**_ **O**_**3**_ **/FeAC/CFP** may originate from its higher total catalytic activity and larger number of accessible Fe sites. The Faradaic efficiencies for NH_3_, nitrite, and HER at different applied potentials are shown in Figure 4. **FeAC/CFP** achieves its highest NH_3_ Faradaic efficiency of 79.6 ± 2.2% at −0.8 V vs RHE, while **Fe**_**2**_ **O**_**3**_ **/FeAC/CFP** reaches an NH_3_ Faradaic efficiency of 37.8 ± 3.1% at the same potential. The electrocatalytic performance of **FeAC** and **Fe**_**2**_ **O**_**3**_ **/FeAC** is compared with that of representative Fe/C-based catalysts reported in the literature (Table S3). For both Fe-containing catalysts, the NH_3_ Faradaic efficiency initially increases with increasing cathodic potential and then decreases at more negative potentials, accompanied by an increase in HER contribution. HER tends to dominate with the increase of cathodic potential, which may be attributed to the lower kinetic barrier for HER relative to the nitrate reduction reaction. Compared to **Fe**_**2**_ **O**_**3**_ **/FeAC/CFP**, **FeAC/CFP** exhibits lower HER Faradaic efficiency and higher NH_3_ selectivity over the investigated potential range. These results suggest that highly dispersed Fe and FeO*_x_* nanoclusters may be more favorable for stabilizing nitrate reduction intermediates and suppressing competitive HER. In contrast, the presence of Fe_2_O_3_ nanoparticles appears to increase HER activity, leading to reduced nitrate reduction selectivity despite the higher overall NH_3_ production rate. Although **Fe**_**2**_ **O**_**3**_ **/FeAC** exhibits higher apparent activity, the intrinsic selectivity toward NH_3_ is compromised due to enhanced HER.

**Figure 4:**
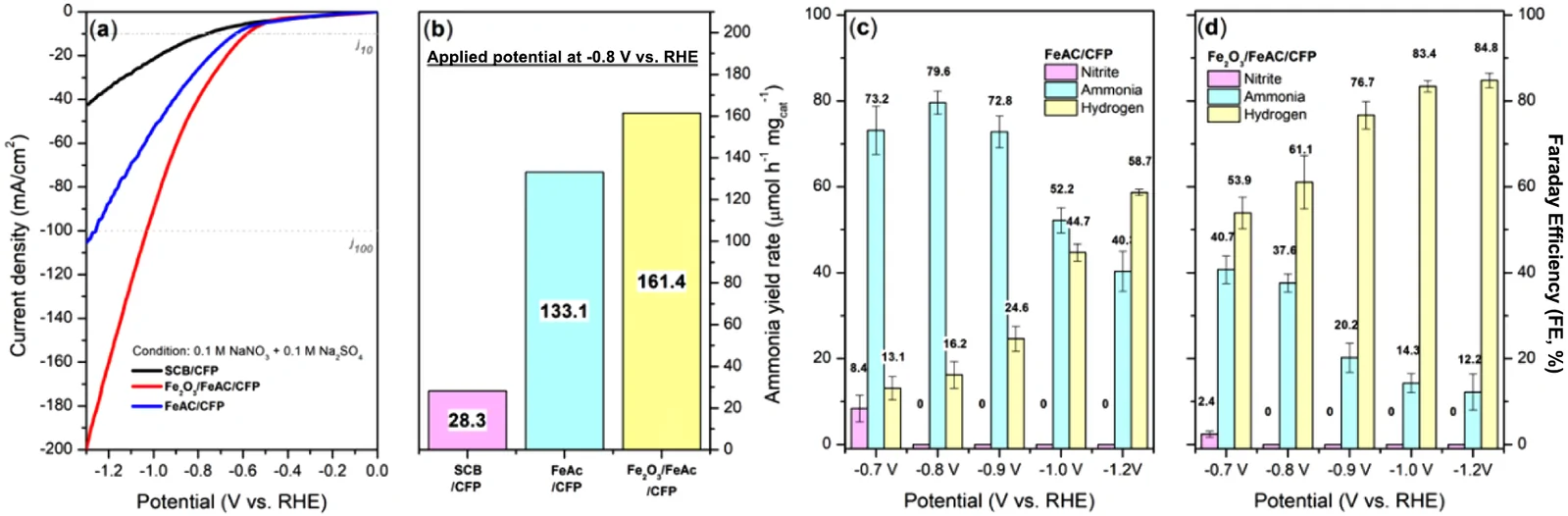
Electrochemical nitrate reduction performance of **FeAC/CFP** and **Fe_2_O_3_/FeAC/CFP** in 0.1 M NaNO_3_ + 0.1 M Na_2_SO_4_ electrolyte. (a) Linear sweep voltammetry curves recorded under nitrate reduction conditions. (b) Ammonia yield rates of the different catalysts at −0.8 V vs RHE. Faradaic efficiencies toward ammonia, nitrite, and hydrogen at different applied potentials for (c) **FeAC/CFP** and (d) **Fe_2_O_3_/FeAC/CFP**.

To evaluate whether the catalytic performance difference originates from variations in electrochemically accessible surface area, the electrochemical double-layer capacitance (*C*_dl_) of **FeAC** and **Fe**_**2**_ **O**_**3**_ **/FeAC** was estimated by cyclic voltammetry in the non-Faradaic region using catalyst-coated carbon fiber paper electrodes in 0.1 M Na_2_SO_4_ (Figure S7). The *C*_dl_ values were determined from the linear relationship between the capacitive current density (Δ*j* = (*j*_a_ – *j*_c_)/2) and scan rate. **FeAC** and **Fe**_**2**_ **O**_**3**_ **/FeAC** exhibited comparable *C*_dl_ values of 16.2 and 15.1 mF cm^–2^, respectively, indicating similar electrochemically accessible interfacial areas. This trend is consistent with previous reports on FeN_*x*_- and Fe cluster/FeN_*x*_-based catalysts, where comparable *C*_dl_ values were also observed despite distinct Fe dispersion states.^28^ These results suggest that the distinct nitrate reduction performance is unlikely to originate primarily from differences in electrochemically accessible surface area but is more reasonably associated with the different Fe dispersion states and the resulting interfacial reaction environments. To further compare the interfacial electrochemical properties of the catalysts under cathodic polarization, electrochemical impedance spectroscopy (EIS) measurements were performed at −0.8 V vs RHE in 0.1 M Na_2_SO_4_ (Figure S8). The impedance spectra were satisfactorily fitted using a two-time-constant Voigt model, and the corresponding fitting parameters are summarized in Figure S8. **FeAC** and **Fe**_**2**_ **O**_**3**_ **/FeAC** exhibited comparable low-frequency charge-transfer resistances (*R*_ct_; 48.67 and 45.54 Ω, respectively), while a slightly higher high-frequency resistance (*R*_1_) was obtained for **Fe**_**2**_ **O**_**3**_ **/FeAC.** These results indicate that the overall interfacial charge-transfer characteristics of the two catalysts are similar under the applied cathodic potential. Taken together, the comparable *C*_dl_ and *R*_ct_ values indicate that neither the electrochemically accessible interfacial area nor the overall interfacial charge-transfer characteristics differ substantially between the two catalysts. Therefore, the observed differences in nitrate reduction performance are more likely associated with differences in Fe-species distribution and the resulting local interfacial reaction environment.

To gain further insight into the origin of the different nitrate reduction selectivity between **FeAC** and **Fe**_**2**_ **O**_**3**_ **/FeAC**, *operando* Raman spectroscopy was employed to probe the evolution of surface species during electrochemical nitrate reduction. As shown in Figure S9, characteristic bands at approximately 910 and 1049 cm^–1^ were consistently observed and assigned to sulfate and nitrate species in the electrolyte, respectively. Upon applying cathodic potentials at −0.8 V vs RHE for 5 min, an additional broad feature centered at approximately 1325 cm^–1^ gradually appeared on both catalysts, suggesting the formation of adsorbed NOx-related intermediates during nitrate reduction. In contrast, only **FeAC** exhibited weak features in the 1445–1500 cm^–1^ region at −0.8 V versus RHE, which are tentatively assigned to nitrate- and NH_*x*_-related surface species.^54–56^ Although the Raman signals are relatively weak because of the low Fe loading and carbon-supported nature of the **FeAC** catalysts, these observations are consistent with the accumulation of nitrogen-containing intermediates under cathodic polarization.

To verify that ammonia originated from nitrate reduction, isotope-labeling experiments were performed using ^15^NO_3_^–^ as the nitrogen source. As shown in Figure S10, electrolysis in the ^15^NO_3_^–^ electrolyte produced the characteristic ^15^NH_4_^+^ doublet arising from ^15^N (*I* = 1/2) coupling, while electrolysis in the ^14^NO_3_^–^ electrolyte gave the corresponding ^14^NH_4_^+^ resonance. In contrast, no ammonium signal was detected in either the open-circuit control or the electrolyte in the absence of nitrate, confirming that NH_4_^+^ was generated exclusively through electrochemical nitrate reduction. In addition to the characteristic ^14/15^NH_4_^+^ resonances, weak adjacent resonances with an interval of approximately 0.02 ppm were observed in both electrolyzed samples (Figure S10). These features are consistent with previously reported H/D isotopologues of ammonium in H_2_O/D_2_O mixtures formed through H/D exchange during sample preparation and NMR measurements.^57–59^ Additionally, GC-MS analysis of the gaseous products revealed the absence of ^15^N_2_ formation after electrolysis in 0.1 M Na_2_SO_4_ + Na^15^NO_3_ (Figure S11).

*Operando* attenuated total reflection-surface enhanced infrared absorption spectroscopy (ATR-SEIRAS) was further employed to investigate hydrogen-related surface species on **Fe**_**2**_ **O**_**3**_ **/FeAC** and **FeAC** in 0.1 M Na_2_SO_4_ electrolyte under different applied potentials (Figure 5). For **Fe**_**2**_ **O**_**3**_ **/FeAC** in H_2_O-based electrolyte, a weak band centered at approximately 1900–2000 cm^–1^ gradually appears with increasing cathodic potential, which is assigned to surface Fe–H species.^60^ Simultaneously, the O–H bending and stretching bands originating from the H_2_O solvent around 1550–1660 and 3000–3600 cm^–1^ vary with increasing cathodic potential, likely reflecting changes in interfacial water ordering and the hydrogen-bonding environment under the applied electric field. Upon replacing H_2_O with D_2_O, the 1900–2000 cm^–1^ feature disappears, and a new band emerges at approximately 1450–1550 cm^–1^, consistent with the expected isotopic shift from Fe–H to Fe–D stretching vibrations. The O–D bending and stretching bands originating from the D_2_O solvent are likewise observed at lower wavenumbers as expected from isotopic substitution. The isotopic shifts, the O–D bending and stretching featuring in the 1150–1250 cm^–1^ and 1900–2000 cm^–1^ region, respectively, assigned to interfacial water instead of other adsorbates.^61,62^ In contrast, **FeAC** mainly exhibits H–O–H bending and O–H stretching frequencies, while no obvious Fe–H-related vibration is observed over the investigated potential range. The absence of detectable Fe–H stretching feature for **FeAC** suggests that hydrogen adsorption is less favorable on the highly dispersed low-coordination Fe species. In contrast, **Fe**_**2**_ **O**_**3**_ **/FeAC** surfaces appear more favorable for the formation of adsorbed hydrogen species under cathodic polarization, which may contribute to enhance HER activity and to decrease nitrate reduction selectivity.

**Figure 5:**
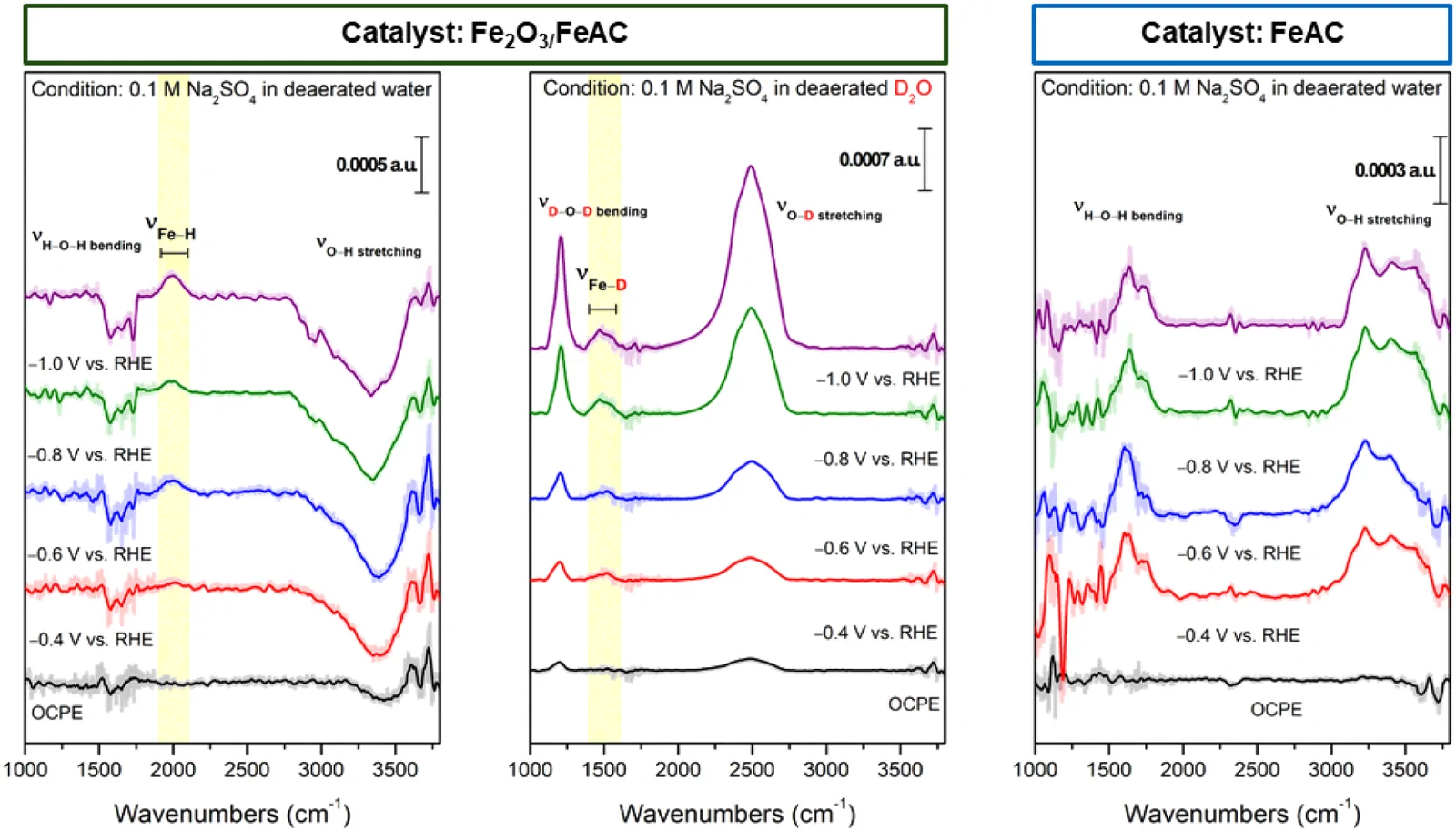
*Operando* ATR-FTIR spectra collected at different applied potentials for **Fe_2_O_3_/FeAC** and **FeAC** in 0.1 M Na_2_SO_4_ electrolyte under deaerated conditions. For **Fe_2_O_3_/FeAC**, spectra recorded in H_2_O and D_2_O reveal potential-dependent vibrational features associated with adsorbed hydrogen species (Fe–H/Fe–D), O–H/O–D bending, and O–H/O–D stretching modes. In contrast, **FeAC** shows no detectable Fe–H-related feature under comparable conditions, indicating less favorable hydrogen adsorption on the highly dispersed Fe sites.

Electron paramagnetic resonance (EPR) measurements using 5,5-dimethyl-1-pyrroline N-oxide (DMPO) as a spin-trapping agent were further carried out after electrolysis at −0.80 V vs RHE with a total charge of 7 C to probe hydrogen-related radical species generated during electrochemical reduction. As shown in Figure 6, **Fe**_**2**_ **O**_**3**_ **/FeAC/CFP** exhibits a significantly stronger DMPO-H signal intensity than **FeAC/CFP** under identical electrochemical conditions, indicating a greater tendency for hydrogen-related radical formation on the iron oxide NPs-containing surface. Simulation of the EPR spectrum for **Fe**_**2**_ **O**_**3**_ **/FeAC/CFP** suggests that the dominant signal can be assigned to DMPO-H radical adducts with *g* = 2.002 and hyperfine coupling constants of *a*_(N,NO)_ = 16.4 G and *a*_(H,CH2)_ = 22.6 G,^63–65^ accounting for approximately 87% of the total signal intensity. A minor contribution at *g* = 2.002 with *a*_(N,NO)_ = 14.6 G can be assigned to a commercially sourced impurity DMPO-centered radical adduct signal,^66^ accounting for the remaining 13% (Figure 6). The much weaker DMPO-H signal observed for **FeAC/CFP** suggests that the highly dispersed Fe species are less favorable for hydrogen adsorption and hydrogen-related radical generation. These results are consistent with the *in operando* IR observations and electrochemical Faradaic efficiency results, supporting the conclusion that iron oxide nanoparticles are associated with increased HER-related activity, while atomically dispersed Fe species in **FeAC** are more selective toward nitrate reduction.

**Figure 6:**
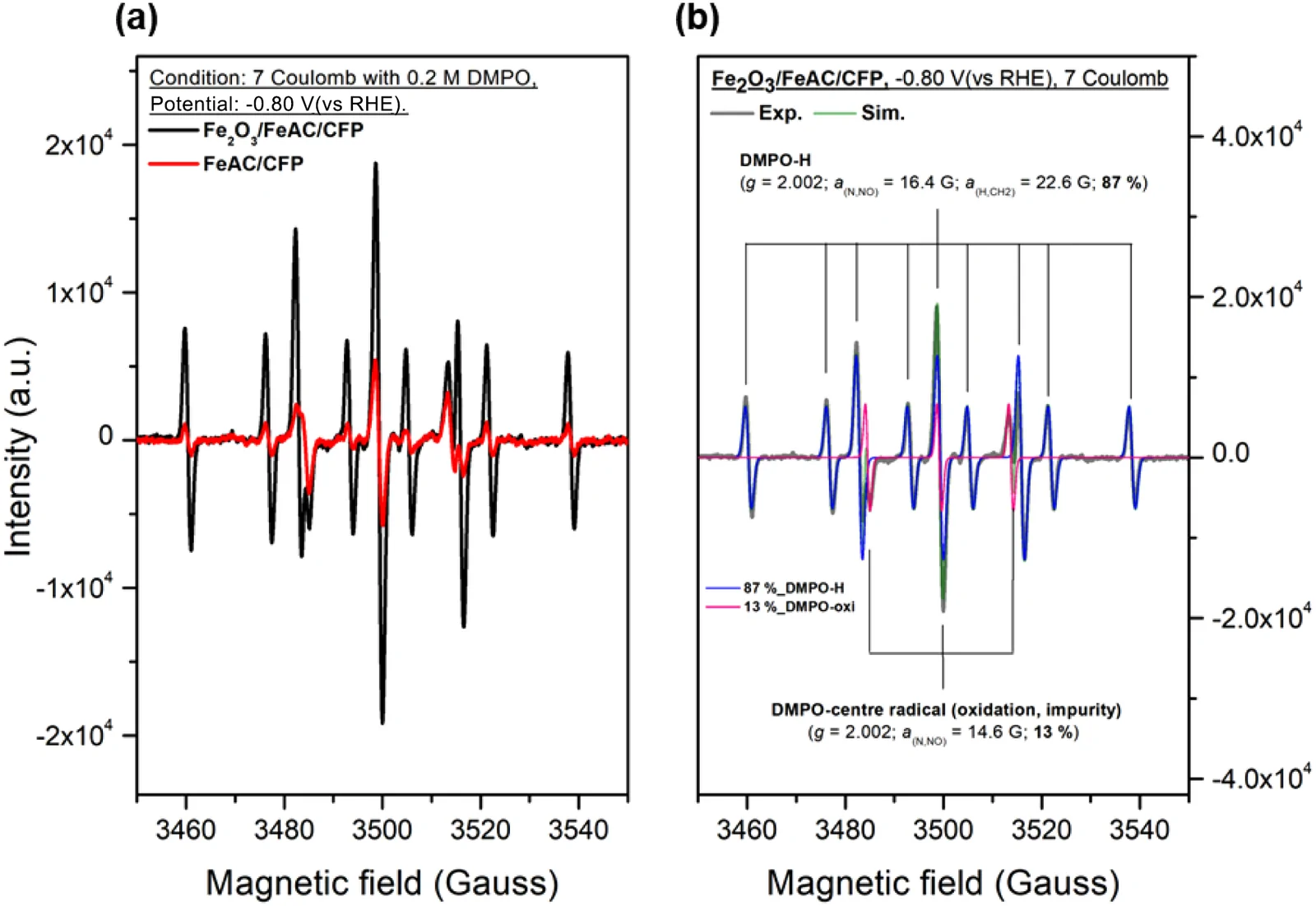
(a) EPR spectra of **Fe_2_O_3_/FeAC/CFP** and **FeAC/CFP** recorded after electrolysis at −0.80 V vs RHE with 0.2 M DMPO as the spin-trapping agent. (b) Simulated fitting of the EPR spectrum for **Fe_2_O_3_/FeAC/CFP**, showing that the dominant trapped species is DMPO–H, together with a minor contribution from a DMPO-centered radical oxidation impurity originating from the purchased DMPO reagent.

To evaluate the structural stability of the highly dispersed Fe species in **FeAC** and nanoparticle-containing **Fe**_**2**_ **O**_**3**_ **/FeAC** during nitrate reduction, postelectrolysis (**post-FeAC** and **post-Fe**_**2**_ **O**_**3**_ **/FeAC**) characterization was performed after 10 h of electrolysis at −0.8 V vs RHE (Figures S12 and S13). Chronoamperometric measurements for both **FeAC/CFP** and **Fe**_**2**_ **O**_**3**_ **/FeAC/CFP** show a gradual decrease in current density during the initial stage, followed by a relatively stable response over prolonged electrolysis. This behavior is likely associated with surface conditioning processes, including electrolyte adsorption and interfacial restructuring, rather than rapid catalyst degradation. The LSV curves recorded before and after electrolysis reveal no significant change in the overall electrochemical response for either catalyst (Figures S12 and S13). While **FeAC/CFP** exhibits a slight decrease in current density after prolonged electrolysis, **Fe**_**2**_ **O**_**3**_ **/FeAC/CFP** shows a slight increase in cathodic current, which may be associated with limited surface reconstruction during electrolysis. ICP-MS analysis revealed decreases in Fe loading from 0.88 ± 0.05 to 0.79 ± 0.03 wt % for **FeAC** and from 1.52 ± 0.04 to 1.38 ± 0.06 wt % for **Fe**_**2**_ **O**_**3**_ **/FeAC**, indicating partial Fe loss during electrolysis (Table S2). Powder X-ray diffraction patterns of **post-FeAC** (Figure S14) showed no newly emerged crystalline FeO_*x*_ or metallic Fe phases, suggesting that no obvious long-range aggregation of Fe species occurred during electrolysis. In contrast, although the characteristic diffraction peak of α-Fe_2_O_3_ were still observed for **post-Fe**_**2**_ **O**_**3**_ **/FeAC**, their intensities were noticeably attenuated after electrolysis, suggesting a decrease in the crystallinity of the Fe_2_O_3_ phase. Nevertheless, AC-TEM and SEM-EDX observations showed no obvious changes in the overall morphology or particle size distribution of the Fe_2_O_3_ nanoparticles (Figures S15–S20), while HRTEM images confirmed that crystalline domains remained observable. Furthermore, HAADF-STEM images of **post-FeAC** and **post-Fe**_**2**_ **O**_**3**_ **/FeAC** still exhibited sparsely distributed atomic-scale bright features on the carbon support, consistent with the presence of highly dispersed Fe species after electrolysis. Collectively, these observations suggest only limited structural evolution during electrolysis despite partial Fe dissolution.

## Conclusion

In this work, the effect of Fe-species distribution on electrochemical nitrate reduction was investigated by comparing **FeAC**, containing highly dispersed Fe species with FeO*_x_* nanoclusters, and **Fe**_**2**_ **O**_**3**_ **/FeAC**, containing additional crystalline α-Fe_2_O_3_ nanoparticles on a carbon support. Structural characterization confirmed the coexistence of isolated atomic-scale Fe species and FeO*_x_* nanoclusters in **FeAC**, whereas **Fe**_**2**_ **O**_**3**_ **/FeAC** additionally contained crystalline α-Fe_2_O_3_ nanoparticles. Electrochemical measurements revealed that **FeAC** achieved a significantly higher NH_3_ Faradaic efficiency (81.4%) at −0.8 V versus RHE than **Fe**_**2**_ **O**_**3**_ **/FeAC** (37.8%), whereas **Fe**_**2**_ **O**_**3**_ **/FeAC** exhibited a higher NH_3_ production rate. This trade-off suggests that Fe-species aggregation promotes overall activity at the expense of reaction selectivity. The comparable electrochemically accessible surface areas and interfacial charge-transfer characteristics indicate that the observed catalytic differences are unlikely to arise primarily from differences in electrochemically accessible surface areas or overall charge-transfer properties. Instead, operando spectroscopic analyses indicate that Fe_2_O_3_ nanoparticle-containing surfaces promote the formation of hydrogen-related Fe–H intermediates and increase HER competition, whereas highly dispersed Fe species suppress hydrogen adsorption while favoring nitrate reduction. These results demonstrate that controlling Fe-species distribution provides an effective strategy for tuning the activity–selectivity relationship of carbon-supported Fe electrocatalysts for electrochemical nitrate reduction.

## Experimental Section

All air-sensitive manipulations, reactions, and transfers were conducted under a pure nitrogen atmosphere according to Schlenk techniques or in a glovebox (N_2_ atmosphere). Solvents were purified and distilled under nitrogen by utilizing suitable reagents (methanol with CaO) and stored in dried, N_2_-filled flasks over 3 Å molecular sieves. Poly­(*o*-phenylenediamine) (PoPDA) and activated carbon (a-CB) were synthesized by the literature procedures. The reagents *o*-phenylenediamine (Sigma-Aldrich), sodium nitrate (Sigma-Aldrich), sodium sulfate (Sigma-Aldrich), sodium hydroxide (Sigma-Aldrich), sodium salicylate (Sigma-Aldrich), sodium nitroprusside (Sigma-Aldrich), sodium dichloroisocyanurate dihydrate (Sigma-Aldrich), Na_2_EDTA (Sigma-Aldrich), thiourea (Sigma-Aldrich), Nafion dispersion solution D2020CS (The Chemours Company), and iron­(III) chloride (Sigma-Aldrich) were used as received. Infrared spectra (IR) were recorded on a PerkinElmer Frontier with sealed solution cells (0.1 mm, KBr window). UV–vis spectra were recorded with an Agilent 8453 spectrometer in a septum-sealed 1 cm pathlength gas-tight quartz cuvette, and all reactions were carried out under a nitrogen atmosphere. ^1^H NMR spectra were obtained on a Varian Unity-700 MHz spectrometer. Analyses of carbon, hydrogen, and nitrogen were obtained with a CHN analyzer (Elementar Vario EL III CHN-OS Rapid). Gaseous products were detected by Shimadzu GC-2030 gas chromatography, equipped with a barrier discharge ionization detector (BID). Powder X-ray diffraction (PXRD), morphologies (FESEM-EDX), high-angle annular dark-field scanning transmission electron microscopy (HAADF-STEM), and X-ray photoelectron spectroscopy (XPS) of Fe/C-based materials (**FeAc** and **Fe**_**2**_ **O**_**3**_ **/FeAc**) were performed as follows . The PXRD pattern collections were performed in Rigaku Smartlab powder X-ray diffractometer system (Cu Kα, λ = 0.154060 nm). The morphologies of resulting Fe/C-based material were loaded on Si substrate and imaged by FESEM (JEOL JSM-7000F equipped with an Oxford EDX system) and HAADF-STEM (JEOL JEM-ARM200FTH (NTHU) and JEM-ARM200F (NCTU)) equipped with an Oxford EDX system, operating at an accelerating voltage of 200 keV, dark-field lattice image resolution: 0.08 nm. XPS measurements of resulting iron-based particles were loaded on Si substrate and conducted on a ULVAC-PHI XPS spectrometer with a monochromatic Al anode as the X-ray source. The XPS depth profile of Fe/C-based material was carried out using 5 kV argon bombardment with a sputter rate of 50.7 nm/min for Fe. XPS data analysis and the peak deconvolution using Gaussian–Lorentzian curve fitting based on Shirley background correction was accomplished with OriginPro 8.5.

### Synthesis of the Iron-Free Catalyst (Thiourea-Treated Active Carbon Black, SCB)

Activated carbon black (a-CB) was prepared by dispersing 1.0 g carbon black in 50 mL 9 M HNO_3_ solution, followed by refluxing with vigorous stirring at 90 °C for 3 h, subsequently washing water/ethanol/ether and then drying under vacuum. Then, a-CB (0.5 g) was dispersed in the 25 mL aqua solution containing thiourea (50 mg) and then transferred into a 25 mL Teflon-lined autoclave with steel shell at 160 °C for 8 h. After mixing, the suspension was filtered to collect the product, washed with water (twice), ethanol, and then dried under vacuum. The as-prepared PoPDA precursor was mixed homogeneously with activated carbon black with a mass ratio of 1:1 and then annealed in a tubular furnace at 700 °C for 4 h under an inert atmosphere with N_2_ flow rate of 0.2 mL min^–1^. After thermal treatment, the resulting black powder (**SCB**) was stored in N_2_-filled glovebox.

### Synthesis of the Atomic-Scale Iron Catalyst (**FeAC**)

A suspension was prepared by mixing 200 mg of PoPDA with 20 mL of a 0.02 mM FeCl_3_ solution in methanol containing 5% water, followed by ultrasonication for 1 h and vigorous stirring for 24 h at 60 °C. The resulting suspension was filtered to collect the product (**Fe/PoPDA**), washed with water (twice), ethanol, and then dried under vacuum. The as-prepared **Fe/PoPDA** precursor (40 mg) was mixed homogeneously with **SCB** (40 mg) with a mass ratio of 1:1 and then annealed in a tubular furnace at 700 °C for 4 h under inert atmosphere with N_2_ flow rate of 0.2 mL min^–1^. The obtained product **FeAC** (32 mg) was stored in N_2_-filled glovebox for characterization. The iron content in **FeAC** was determined to be 0.77 ± 0.04 ppm (0.88 ± 0.05 wt %) by ICP-MS. The sulfur and nitrogen contents in **FeAC** were determined to be 1.92 and 4.89 wt %, respectively, by elemental analysis.

### Synthesis of Iron Oxide-Decorated Atomic-Scale Iron Catalysts (**Fe_2_O_3_/FeAC**)

A suspension was prepared by mixing 200 mg of PoPDA with 20 mL of a 0.1 mM FeCl_3_ solution in methanol containing 5% water, followed by ultrasonication for 1 h and vigorous stirring for 24 h at 60 °C. The resulting suspension was filtered to collect the product (**Fe/PoPDA**), washed with water (twice), ethanol, and then dried under vacuum. The as-prepared **Fe/PoPDA** precursor (40 mg) was mixed homogeneously with **SCB** (40 mg) with a mass ratio of 1:1 and then annealed in a tubular furnace at 700 °C for 4 h under inert atmosphere with N_2_ flow rate of 0.2 mL min^–1^. The obtained composite material **Fe**_**2**_ **O**_**3**_ **/FeAC** (52 mg) was stored in N_2_-filled glovebox for characterization. The powder XRD pattern of **Fe**_**2**_ **O**_**3**_ **/FeAC** exhibits a characteristic *R*3̅*c* α-Fe_2_O_3_ phase, with reflections at 24.2°, 33.2°, 35.6°, 43.7°, 49.5°, 54.1°, 57.6°, 62.5°, 64.0°, and 72.0 °. The iron content in **Fe**_**2**_ **O**_**3**_ **/FeAC** was determined to be 2.23 ± 0.06 ppm (1.52 ± 0.04 wt %) by ICP-MS. The sulfur and nitrogen contents in **Fe**_**2**_ **O**_**3**_ **/FeAC** were determined to be 3.02 and 8.24 wt %, respectively, by elemental analysis. FESEM images of **Fe**_**2**_ **O**_**3**_ **/FeAC** exhibit roughly spherical particles with size range from 60 to 120 nm.

### Electrode Preparation

Fe/C-based materials (**FeAC** and **Fe**_**2**_ **O**_**3**_ **/FeAC**) and SCB (without Fe loading) were prepared by dispersing 1 mg of catalyst in 40 μL of a isopropanol solution with 20 μL of 5 wt % Nafion, followed by 30 min sonication. A fixed volume of the ink was drop-cast onto a 0.2 cm × 0.4 cm carbon fiber paper (CFP) and dried in air (1.0 mg composite material loading onto 0.1 cm^–2^ substrate).

### Electrochemical Measurements

Electrochemical measurements were performed in a two-compartment H-type cell separated by a PVDF membrane (Merck) using a CHI 611E electrochemical workstation (CH Instruments, Inc.). A carbon rod, saturated calomel electrode (SCE), and catalyst-coated 2D carbon-fiber paper (catalyst loading: 1.0 mg cm^–2^) were employed as the counter, reference, and working electrodes, respectively. All potentials were converted to the reversible hydrogen electrode (RHE) scale according to the following eq 1:

1 E(Vvs.RHE)=E(Vvs.SCE)+0.241+0.0591×pH

Linear sweeping voltammetry, cyclic voltammetry, chronoamperometry, and double-layer capacitance measurements were performed in 0.1 M NaNO_3_ + Na_2_SO_4_ and 0.1 M Na_2_SO_4_ under magnetic stirring. Electrochemical impedance spectroscopy measurements were carried out using a Zahner Zennium ε galvanostatic instrumentation with a standard three-electrode system. To eliminate mass-transfer kinetics, 2D carbon-fiber paper was used as the working electrode to explore interfacial behavior. EIS measurements were conducted at −0.80 V versus RHE over a frequency range from 1000 kHz to 0.1 Hz.

### *Operando* Raman Spectroscopy

*Operando* Raman spectra were collected using a Renishaw inVia confocal Raman microscope equipped with a 532 nm excitation laser. The spectral resolution was 1 cm^–1^, and the Raman shift was calibrated prior to each measurement using the characteristic silicon band at 520.7 cm^–1^. *Operando* Raman measurements were performed in a homemade spectro-electrochemical cell using a catalyst-coated glassy carbon electrode (**FeAC** or **Fe**_**2**_ **O**_**3**_ **/FeAC**) as the working electrode, a Pt wire as the counter electrode, and an SCE as the reference electrode. The electrolyte consisted of 0.1 M NaNO_3_ + 0.1 M Na_2_SO_4_. Raman spectra were first collected under OCP, followed by chronoamperometric electrolysis at −0.8 V versus RHE, during which spectra were continuously recorded as a function of electrolysis time (5 min). To minimize signal attenuation caused by gas evolution during electrolysis, the electrolyte was continuously circulated through the spectro-electrochemical cell throughout the measurements.

### Isotope Labeling Experiments

^1^H NMR analysis of ammonia. Electrolysis was conducted in 0.1 M Na_2_SO_4_ containing 0.1 M Na^14/15^NO_3_ at −0.80 V versus RHE until 30 C. After electrolysis, 0.50 mL of the electrolyte was mixed with 0.05 mL of 1.0 M HCl to convert dissolved NH_3_ into NH_4_.^24,67^ The resulting solution was mixed with 0.05 mL D_2_O for the field-frequency lock, and ^1^H NMR spectra were recorded on a Varian 700 MHz NMR spectrometer. Isotope-labeling experiments were performed using either Na^14^NO_3_ or Na^15^NO_3_ as the nitrate source under identical electrolysis conditions. The same sample preparation procedure was applied to the open-circuit and nitrate-absent control experiments. Additionally, gas chromatography–mass spectrometry (GC–MS) was performed. The gaseous products were analyzed using a high-resolution gas chromatography–mass spectrometer (HRGC–MS, Thermo Scientific Trace 1300 GC coupled with an ISQ-MS) equipped with an RT-Msieve 5 Å capillary column (15 m). Helium was used as the carrier gas at a flow rate of 1.0 mL min^–1^. Isotope-labeling experiments were performed using Na^15^NO_3_ as the nitrogen source to examine the possible formation of ^15^N_2_ during electrochemical nitrate reduction.

### *Operando* Attenuated Total Reflection-Surface Enhanced Infrared Absorption Spectroscopy (ATR-SEIRAS)

Electrochemical ATR-SEIRAS experiments were carried out BL14A at the National Synchrotron Radiation Research Center (NSRRC), Hsinchu, Taiwan. The reflecting plane of a 60° Si prism (Veemax, 2 cm in diameter) was polished with alumina pastes and then with 0.2% hydrofluoric acid for 30 min each to expose fresh Si layer. After drying, the conductive copper tapes were attached around the prism, and the catalyst (**Fe**_**2**_ **O**_**3**_ **/FeAC** and **FeAC**) was deposited on the surface of the Si prism, serving as the working electrode in a spectroelectrochemical cell along with reference saturated calomel electrode (SCE) and Pt-wire counter electrode. The electrochemical cell was integrated into a Fourier transform infrared spectrometer (Thermo Scientific Nicolet 6700 FT-IR), equipped with a mercury cadmium telluride (MCT) detector and a variable-angle specular reflectance accessory (Veemax III, Pike Technologies). Using the baseline spectrum recorded at open-circuit potential, cathodic chronoamperometric scans were performed at incrementally more negative potential, ranging from −0.6 V (vs RHE) to −1.0 V (vs RHE). Electrochemical measurements were carried out at each target potential in 1.0 M Na_2_SO_4_ in H_2_O or D_2_O, using a potentiostat. Each potential was held for 1 min, during which the corresponding IR spectrum was collected. For deuterium isotope experiments, only the solvent with D_2_O to identify the Fe–H/D and N–H/D species. All spectra were recorded in absorbance mode with a spectral resolution of 4 cm^–1^.

### *In Situ* Electron Paramagnetic Resonance Spectroscopy (EPR)

The *in situ* EPR measurements were performed at X-band (9.82 GHz) using a Bruker EMX spectrometer equipped with a Bruker TE102 super-high-sensitivity probe head cavity. 5,5-Dimethyl-1-pyrroline-*N*-oxide (DMPO, 40 mM) was used as a spin trap to identify the water-dissociated H radical at −0.8 V (vs RHE) with various electrodes (**FeAC, Fe**_**2**_ **O**_**3**_ **/FeAC**) in N_2_-saturated 0.1 M Na_2_SO_4_ aqueous electrolyte at ambient temperature. The aqueous samples were loaded into a Suprasil flat quartz cell for the EPR measurement. EPR spectra of DMPO-H and DMPOX derivatives were obtained with a frequency at 9.82 GHz. The microwave power and modulation amplitude were 15.0 mW and 0.8 G at 100.00 kHz, respectively. For the spin-trap experiment, **FeAC** and **Fe**_**2**_ **O**_**3**_ **/FeAC** electrodes were applied at −0.8 V (vs RHE) until achieving 7.0 C prior to the data collection. The carbon-fiber paper electrode was employed as a blank with silence behavior. EPR simulations were carried out by Matlab software with EasySpin.^68^

### Determination of the Liberated NH_3_/NH_4_^+^ by the Indophenol Blue Method

Quantification of ammonia concentration generated from electrochemical nitrate reduction was determined using the indophenol (salicylate-Berthelot) method, in which sodium salicylate served as the phenolic reagent and sodium dichloroisocyanurate dihydrate (NaDCC) was employed as the available chlorine source.^69^ All solutions were prepared with deionized water to minimize background contamination by ambient ammonia. A standard ammonium sulfate solution (1.0 g NH_3_/L; 55.4 mM) was prepared and diluted to a suitable calibration range (0, 0.2, 0.5, 1.0, 2.0 mg NH_3_/L). **Reagent A** was prepared by dissolving 6.906 g sodium salicylate and 0.225 g sodium nitroprusside in 250 mL of 0.5 M NaOH and stored in an amber bottle at 4 °C. **Reagent B** was prepared by dissolving an amount of 0.5 g NaDCC (which is equivalent to the available chlorine content of 7.5 mL of 10–15% NaOCl) in 250 mL of 1.0 M NaOH and stored in an amber bottle at 4 °C. For color development and measurement, 3 mL of the sample solution was mixed with 0.3 mL of **Reagent A**, followed by 0.3 mL of **Reagent B**. The mixture was gently shaken and allowed to stand at room temperature for 70 min in the dark to complete color stabilization. The absorbance was then measured at 630 nm (to minimize noise) using a UV–Vis spectrophotometer with a 1.0 cm optical path. Calibration curves were constructed using standard ammonium solutions, and the ammonia concentration in samples was determined from the linear regression of absorbance versus NH_3_ concentration (Figure S21a). Blank and standard addition tests were conducted to verify accuracy and rule out background interference. The method relies on the *in situ* formation of monochloramine by reaction of ammonia with the active chlorine species released from NaDCC under alkaline conditions, followed by condensation with sodium salicylate in the presence of nitroprusside catalyst to yield an indophenol blue chromophore. The procedure provides a sensitive and reliable means of quantifying trace ammonia produced during electrochemical nitrate reduction.

### Determination of Liberated Nitrite Ions by the Griess Test

Quantification of the released nitrite was completed via the Griess reagent test (the feature peak (absorbance at 540 nm) recorded by an Agilent 8453 UV/vis spectrophotometer). The Griess test solution was prepared by weighing 35 mg of modified Griess reagent powder (Sigma G4410) and then dissolving it with 50 mL of deaerated water.^70^ The calibration curves were derived by various concentrations of sodium nitrite solution (0, 3.3, 8.2, 9.9, 16.5, and 23.0 mM) and were plotted in­(Figure S21b.

### Determination of the Liberated N_2_O Gas by GC-BID

Quantification of NO and N_2_O was performed on Shimadzu GC-2030 gas chromatography equipped with a barrier discharge ionization detector (BID), and Shin Carbon ST (100/120 mesh) columns filled with poly­(dimethyl)­siloxane (stationary phase, Rtx-1). Helium was adopted as mobile phase to conduct sample separation. The oven temperature was kept at 40 °C, then raised up to 240 °C (rate 20 °C/min), and the detector was heated to 280 °C. The standard N_2_O gas exhibited a retention time of 8.2 min. In this report, no N_2_O was detected during the electrocatalytic NO_3_RR.

### X-ray Absorption Spectroscopy (XAS)

X-ray absorption experiments were carried out at the National Synchrotron Radiation Research Center (NSRRC), Hsinchu, Taiwan. Samples were ground to powder and secured in the bag made of 6 μm Mylar film. The Fe K-edge X-ray absorption spectroscopy (XAS) and S K-edge XAS data were collected in fluorescence mode at BL17C (X-ray absorption fine structure spectroscopy, 4.5–13 keV) and BL16A1 (tender X-ray absorption spectroscopy, 1 to 8 keV) with a double crystal Si(111) monochromator. The energy resolution Δ*E*/*E* was estimated to be about 2 × 10^–4^. High harmonics were rejected by Rh-coated mirrors. The energy is scanned from 2.402 to 2.652 keV for S K-edge and from 6.912 to 7.609 keV for Fe K-edge. A Lytle detector was employed for fluorescence measurements. The photon energy was calibrated to the maximum of the first inflection point at 2472.0 eV for the sulfur spectrum and 7112.0 eV for the Fe foil spectrum, respectively. All data are calibrated, analyzed, and fitted using Athena software. Data were averaged, and a smooth background was removed from all spectra by fitting a straight line to the pre-edge region and subtracting this straight line from the entire spectrum. Normalization of the data was accomplished by fitting a flat polynomial to the postregion and normalizing the edge jump to 1.0.^71–75^

## Supplementary Material



## Notes

Additional experimental details and spectra, including photographs of experimental setup (PDF)
