# Supplementary material for: Selectivity of Nitrate Reduction and Hydrogen Evolution Reaction Modulated by Aggregation of Carbon-Supported Fe Species
Source: Langmuir. 2026 Sep 3;42(36):26655–68. doi: 10.1021/acs.langmuir.6c03729 (PMC13585132; doi:10.1021/acs.langmuir.6c03729)
Supplement: Supplementary file 1 [file la6c03729_si_001.pdf]

Supporting Information

Selectivity of Nitrate Reduction and Hydrogen Evolution Reaction Modulated by Aggregation of Carbon-Supported Fe Species

Wun-Yan Wu,*[a] Cheng-Jhe Tsai,[a] Chih-Ying Lin,[a] Serhii Makovetskyi,[a] Ting-Shan Chan,[b] Yao-Chang Lee,[b] Ying-Rui Lu,[b] Wen-Feng Liaw*[a]

[a] Department of Chemistry, National Tsing Hua University, Hsinchu 30013, Taiwan
[b] National Synchrotron Radiation Research Center, Hsinchu 300092, Taiwan

E-mail address:
wywu@gapp.nthu.edu.tw (WYW)
wfliaw@mx.nthu.edu.tw (WFL)

Table of Contents

Figure S1. (a) PXRD patterns of **SCB**, **FeAC**, and **Fe_2O_3/FeAC**, (b) FTIR spectra of the PoPDA precursor, **SCB**, **FeAC**, and **Fe_2O_3/FeAC**. **S3**

Figure S2. SEM images of the **FeAC** **S3**

Figure S3. SEM-EDX mapping of the **FeAC**.......... **S4**

Figure S4. SEM images of the **Fe_2O_3/FeAC** **S4**

Figure S5. SEM-EDX mapping of the **Fe_2O_3/FeAC**.......... **S5**

Figure S6. AC-TEM image of the **Fe_2O_3/FeAC** catalyst and corresponding SAED pattern of the **Fe_2O_3/FeAC** catalyst **S6**

Figure S7. ECSA of **FeAC and Fe_2O_3/FeAC** in 0.1 M Na_2SO_4 .... **S6**

Figure S8. EIS of **FeAC and Fe_2O_3/FeAC** recorded in 0.1 M Na_2SO_4 at −0.8 V vs. RHE.......... **S7**

Figure S9. *Operando* Raman spectra of **FeAC** and **Fe_2O_3/FeAC** recorded in 0.1 M $NaNO_3$ + Na_2SO_4 aqueous electrolyte under OCP and −0.8 V versus RHE. **S8**

Figure S10. ^1H NMR spectra for the identification of $^{14/15}NH_4^+$ generated by electrochemical nitrate reduction. **S9**

Figure S11. GC-MS of the gaseous products collected after electrolysis of **FeAC** at −0.80 V versus RHE in 0.1 M Na_2SO_4 containing $Na^{15}NO_3$. **S10**

Figure S12. LSV curves of **FeAC/CFP**, and **post-FeAC/CFP** in 0.1 M $NaNO_3$ + 0.1 M Na_2SO_4 electrolyte and longevity.......... **S10**

Figure S13. LSV curves of **Fe_2O_3/FeAC/CFP**, and **post- Fe_2O_3/FeAC/CFP** in 0.1 M $NaNO_3$ + 0.1 M Na_2SO_4 electrolyte and longevity **S11**

Figure S14. PXRD patterns of **post-Fe_2O_3-FeAC** and **post-FeAC.** **S11**

Figure S15. AC-TEM image of **post-FeAC** **S12**

Figure S16. AC-TEM image of **post-Fe_2O_3/FeAC** **S13**

Figure S17. SEM images of the **post-FeAC** **S13**

Figure S18. SEM images of the **post-Fe_2O_3/FeAC** **S14**

Figure S19. SEM-EDX mapping of the **post-FeAC** **S15**

Figure S20. SEM-EDX mapping of the **post-Fe_2O_3/FeAC** **S16**

Figure S21. Calibration curve of qualification of liberated NH_3/ NH_4^+ established by indophenol blue method and liberated NO_2^- by reactions of Griess reagent **S17**

Table S1. Experimental details for as-prepared samples.......... **S17**

Table S2. Elemental content of pre-catalysts and post-catalysts analyzed by ICP-MS. **S17**

Table S3. Comparison of NH_3 yield and FE of different Fe/C-based catalysts for electrochemical NO_3RR.......... **S18**

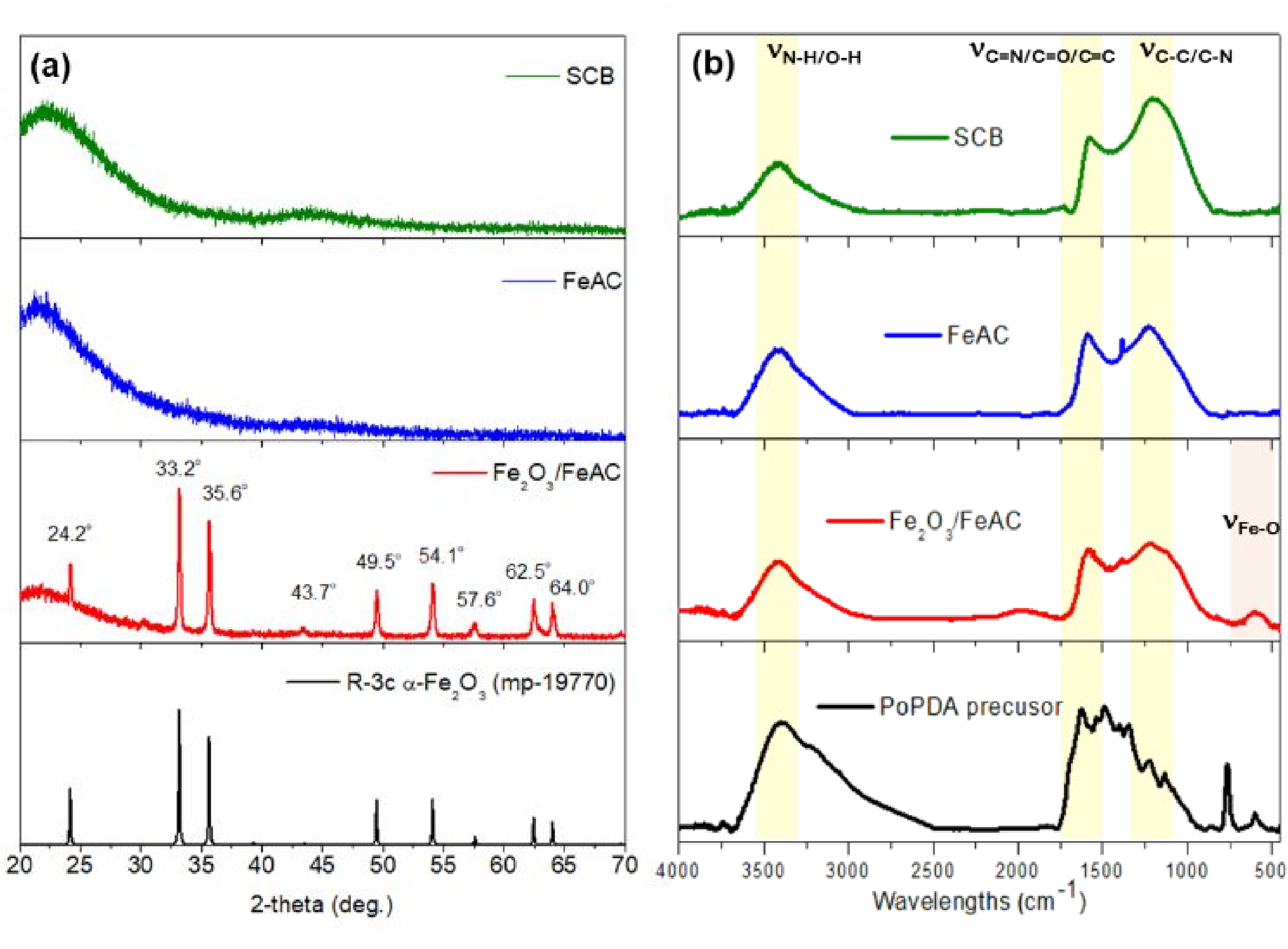

Figure S1. (a) PXRD patterns of **SCB**, **FeAC**, and **Fe_2O_3/FeAC**, together with the reference pattern of rhombohedral α-Fe_2O_3 (R-3c, mp-19770). The diffraction peaks of **Fe_2O_3/FeAC** are consistent with crystalline α-Fe_2O_3.[1] (b) FTIR spectra of the PoPDA precursor, **SCB**, **FeAC**, and **Fe_2O_3/FeAC**.

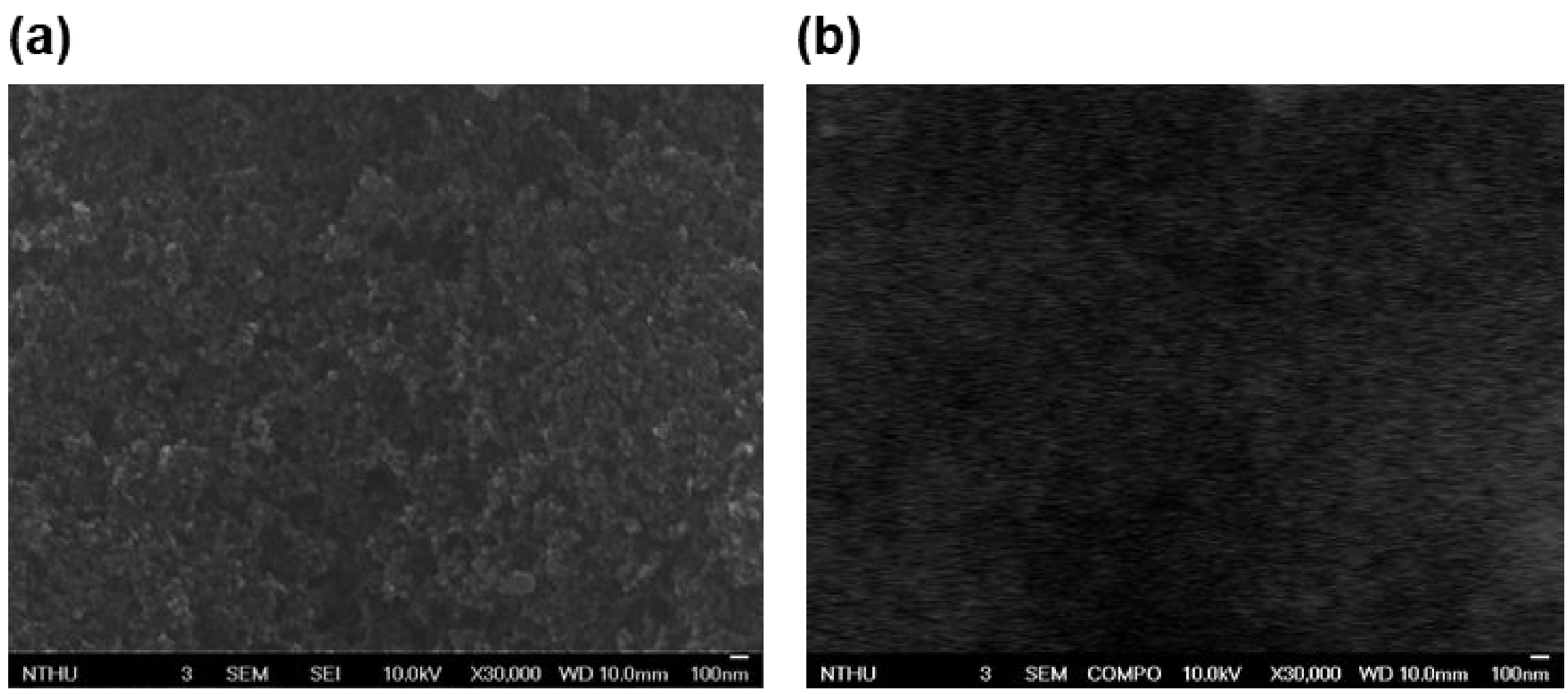

Figure S2. SEM images of the **FeAC** acquired in (a) conventional SE mode and (b) BSE mode.

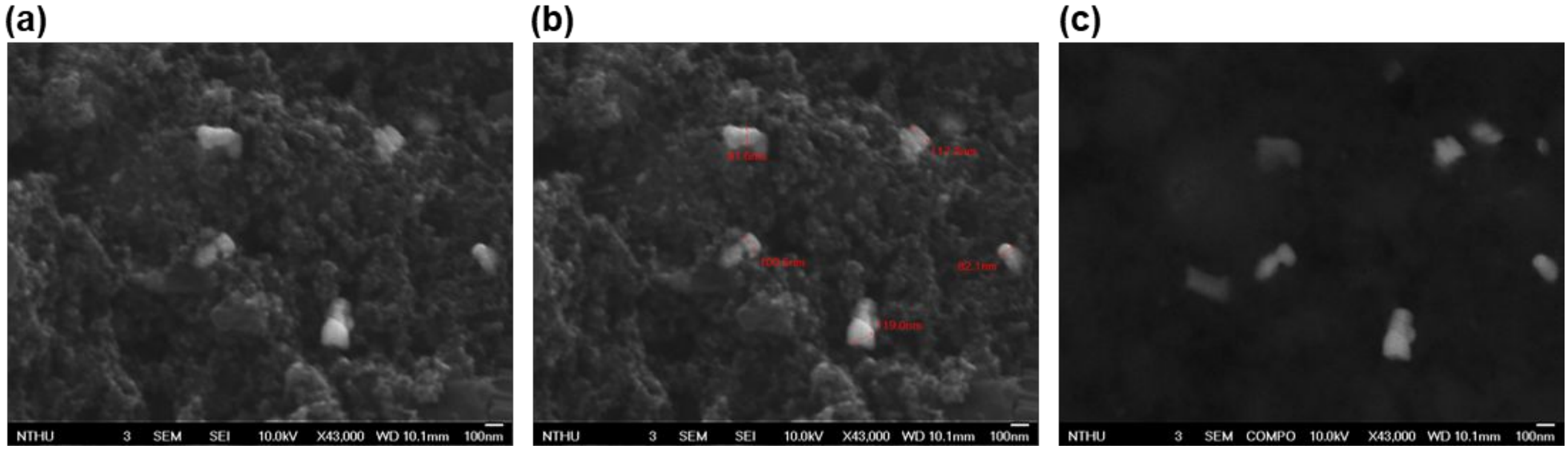

Figure S3. SEM images of the **Fe_2O_3/FeAC** acquired in (a) conventional SE mode with representative feature (b) sizes labeled in the SE image, and (c) BSE mode.

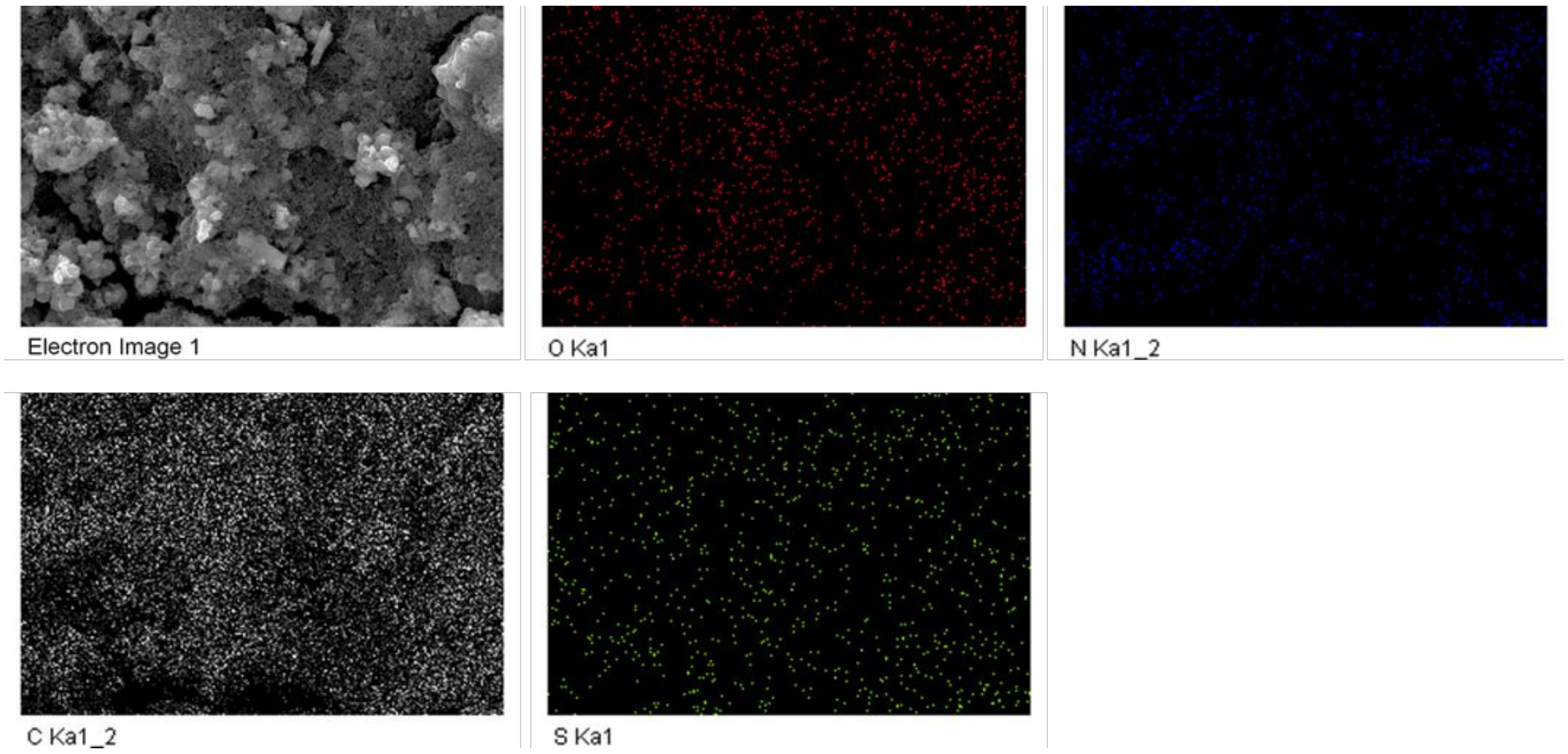

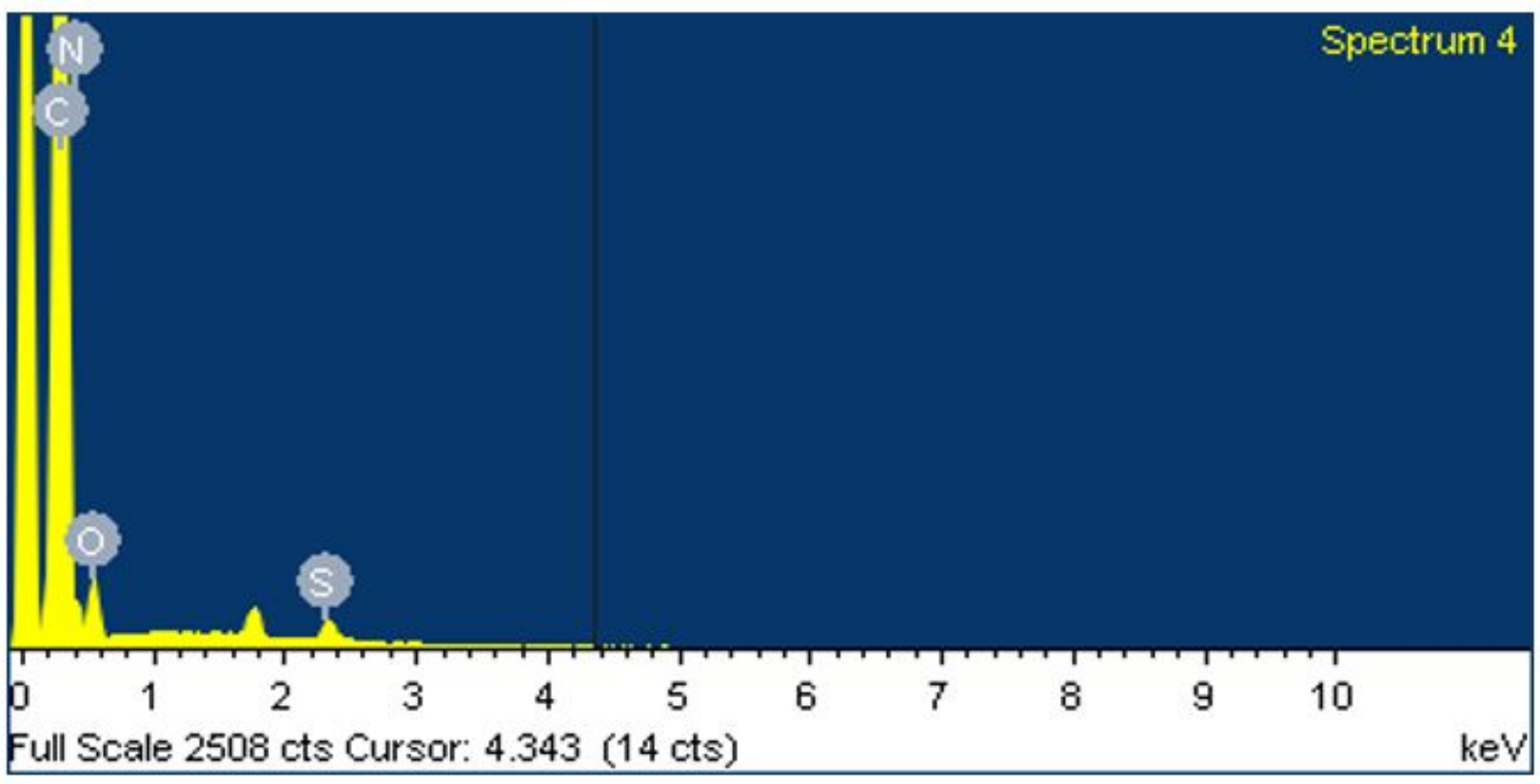

Element	Atomic %	Weight %
C K	86.82	82.97
N K	8.33	9.29
O K	3.63	4.62
S K	1.22	3.11
Fe L	undetectable	undetectable

Figure S4. SEM image, corresponding EDX elemental mapping images (C, N, O, and S), EDX spectrum, and quantitative elemental composition of the **FeAC**. The elemental maps indicate a relatively homogeneous distribution of C, N, O, and S throughout the **FeAC** surface.

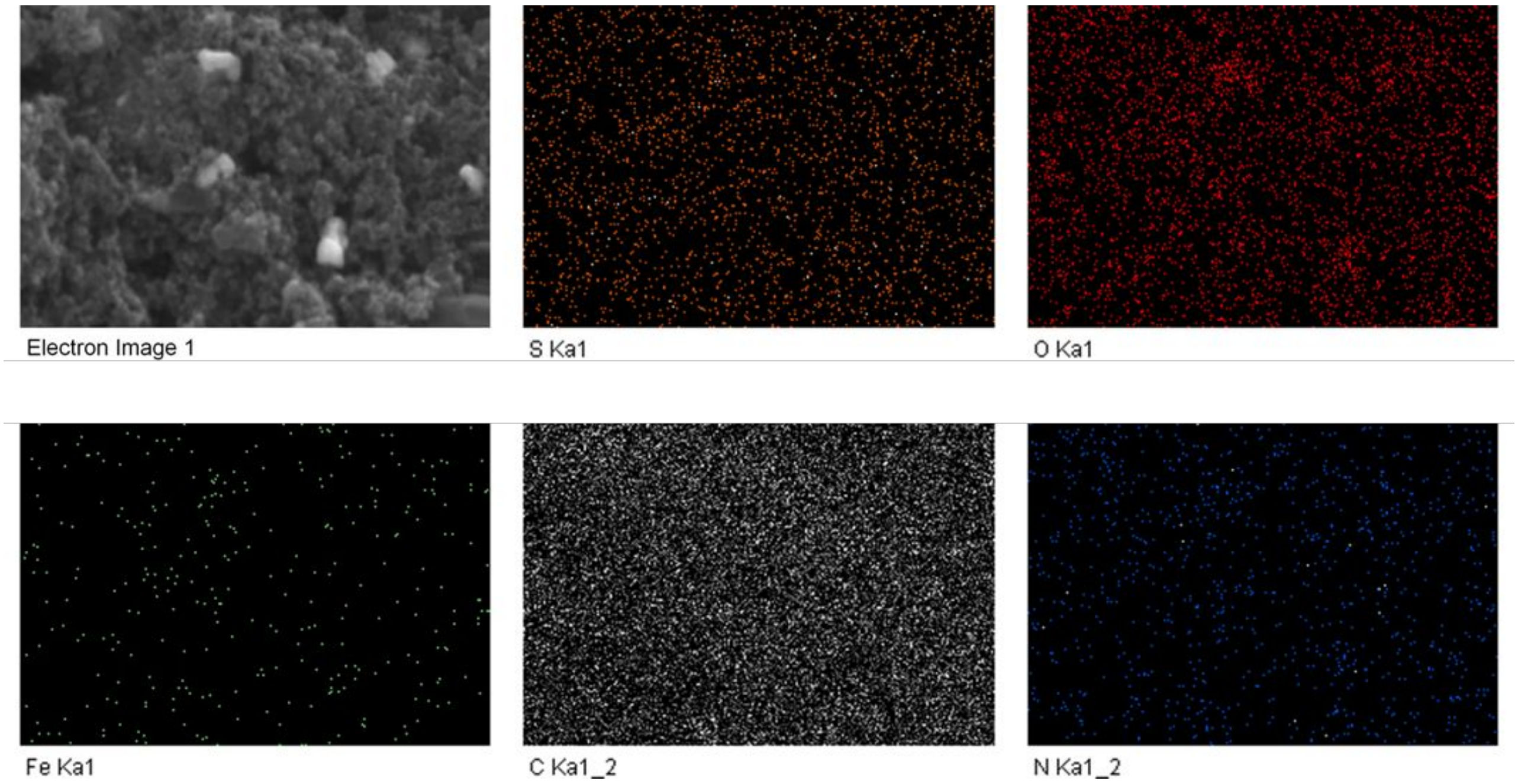

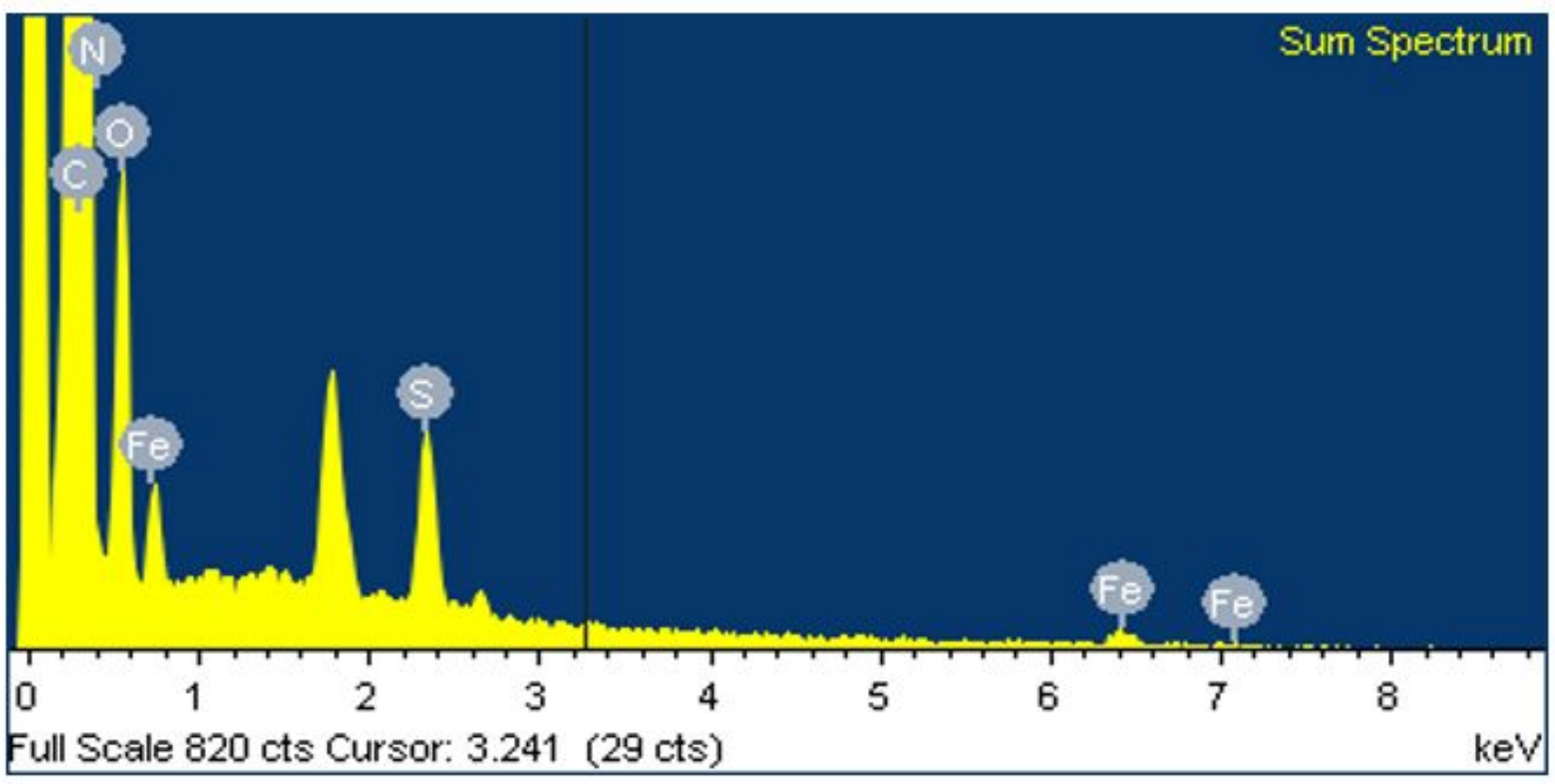

Element	Atomic %	Weight %
C K	83.50	75.31
N K	5.63	5.92
O K	8.35	10.03
S K	0.98	2.36
Fe L	1.52	6.37

Figure S5. SEM image, corresponding EDX elemental mapping images (C, N, O, S and Fe), EDX spectrum, and quantitative elemental composition of the **Fe_2O_3/FeAC**. The elemental maps indicate a relatively homogeneous distribution of C, N, O, S, and Fe throughout the **Fe_2O_3/FeAC** surface.

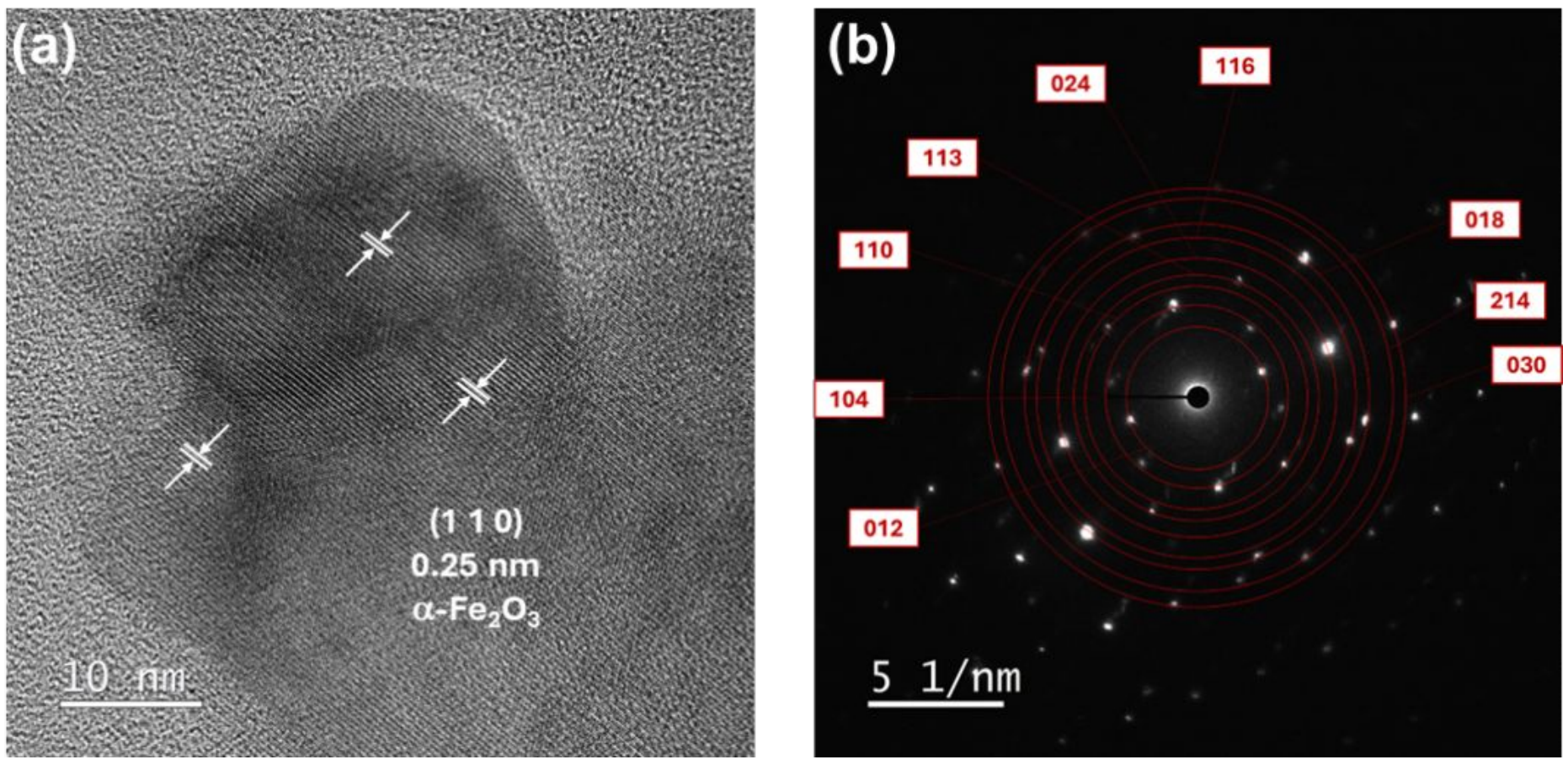

Figure S6. (a) Bright-field AC-TEM image of the **Fe_2O_3/FeAC** catalyst, showing dispersed crystalline α-Fe_2O_3 nanoparticle domains embedded on the carbon support. The observed lattice fringes with interplanar spacings of 0.250 nm are assigned to the (110) plane of α-Fe_2O_3. (b) Corresponding SAED pattern of the **Fe_2O_3/FeAC** catalyst, where the diffraction rings can be indexed to the crystalline α-Fe_2O_3 phase.

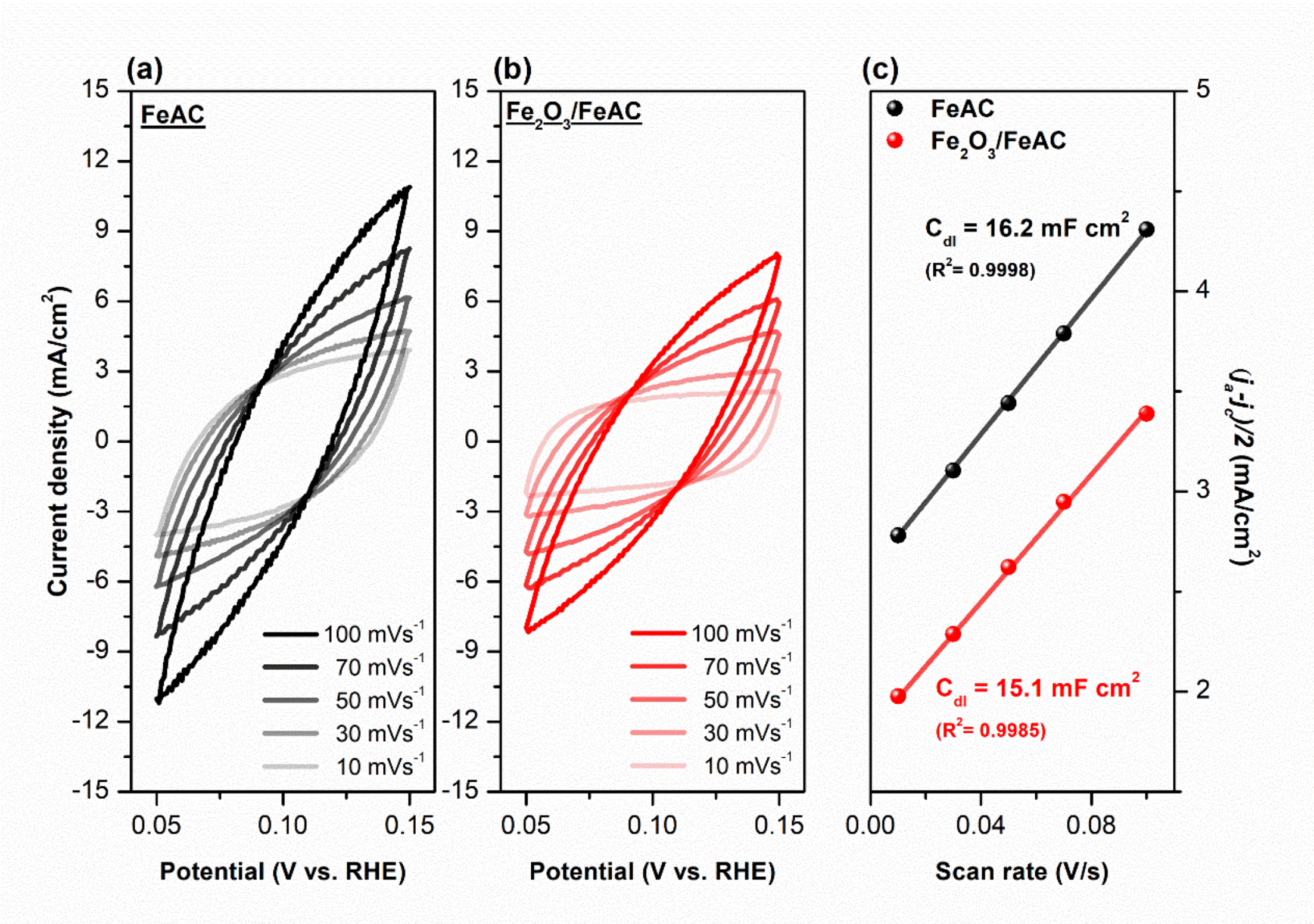

Figure S7. (a) Cyclic voltammograms of **FeAC** recorded in the non-Faradaic potential region at scan rates of 10, 30, 50, 70, and 100 mV s^{-1} in 0.1 M Na_2SO_4 using catalyst-coated carbon fiber paper (CFP) as the working electrode. (b) Cyclic voltammograms of **Fe_2O_3/FeAC** measured under the same conditions. (c) Determination of the double-layer capacitance (C_{dl}) from the linear relationship between the capacitive current density ($\Delta j/2$

= (j_a-j_c)/2) and scan rate, yielding C_{dl} values of 16.2 and 15.1 mF cm^{-2} for **FeAC** and **Fe_2O_3/FeAC**, respectively.

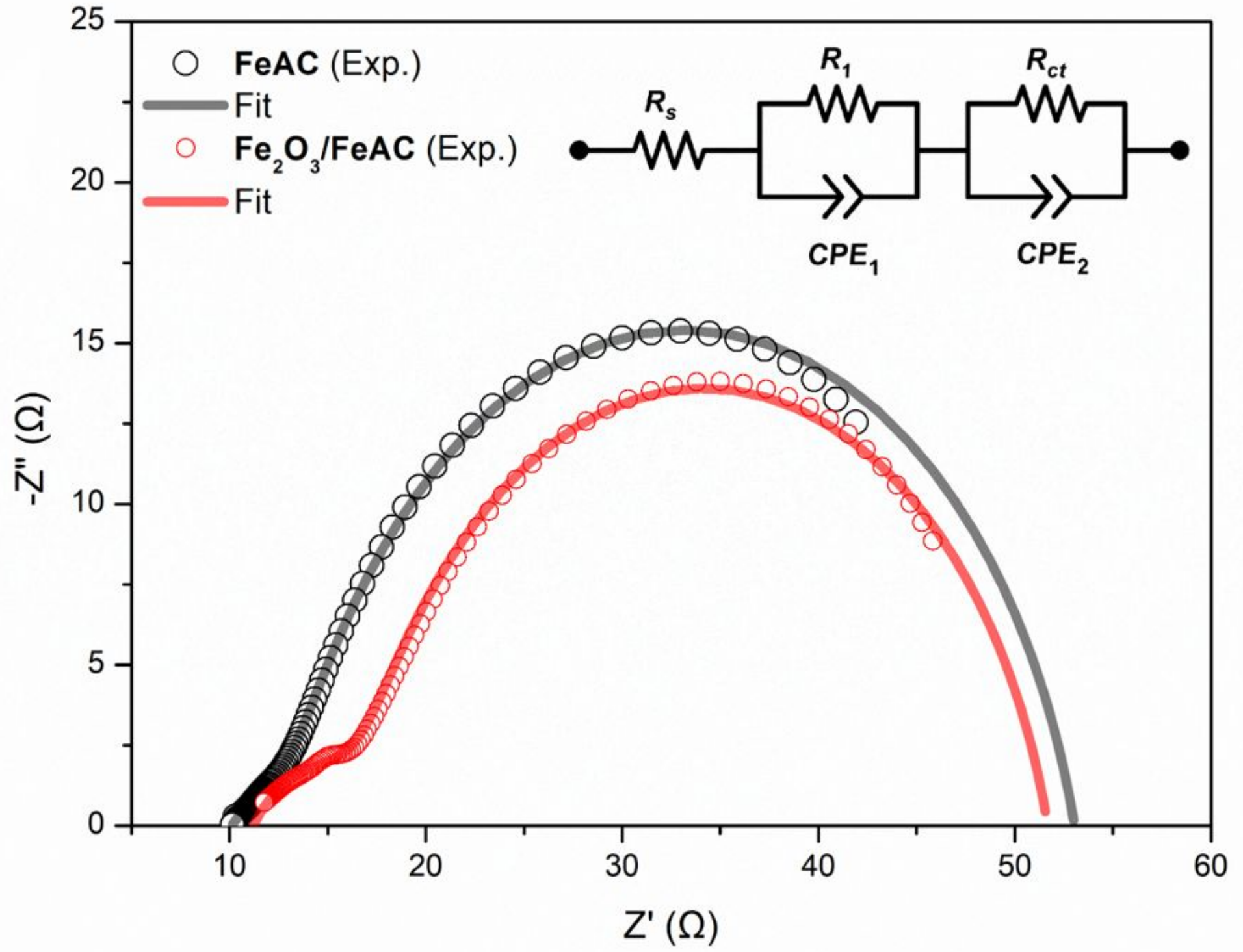

Sample	R_s (Ω)	R_1 (Ω)	R_{ct} (Ω)
**FeAC**	10.24	4.30	48.67
**Fe_2O_3/FeAC**	11.04	6.02	45.54

Figure S8. EIS Nyquist plots of **FeAC** (black)and **Fe_2O_3/FeAC** (red) recorded in 0.1 M Na_2SO_4 at −0.8 V vs. RHE. Open circles represent the experimental data, and solid lines represent the fitted curves. The inset shows the Voigt circuit model used for fitting, and the corresponding fitting parameters (R_s, R_1 and R_{ct}) are summarized in the table below.

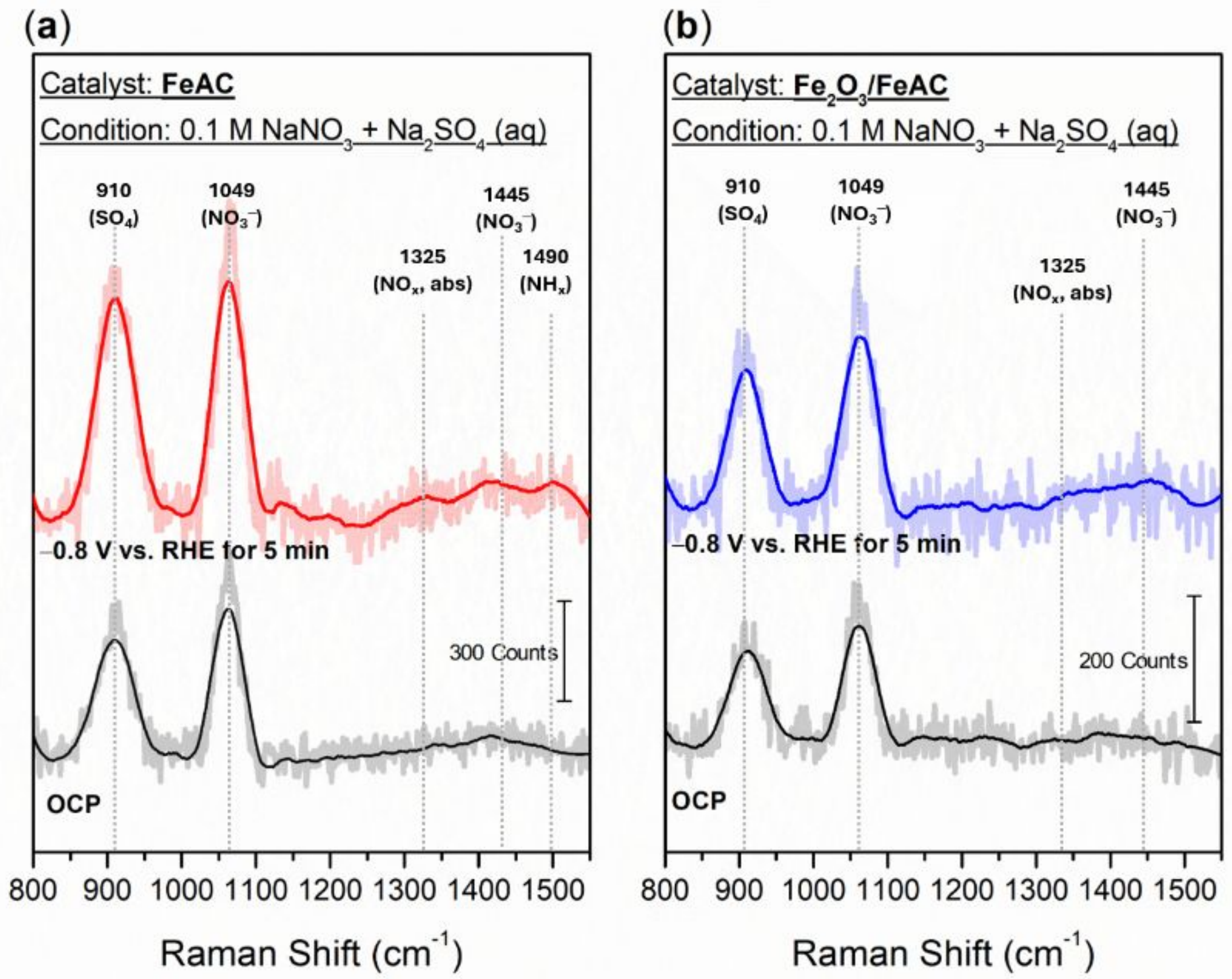

Figure S9. *Operando* Raman spectra of (a) **FeAC** and (b) **Fe_2O_3/FeAC** recorded in 0.1 M $NaNO_3$ + Na_2SO_4 aqueous electrolyte under OCP and −0.8 V versus RHE. Spectra at applied potentials were collected after 5 min electrolysis. The bands at approximately 910 and 1049 cm^{-1} are assigned to SO_4^{2-} and NO_3^-, respectively.

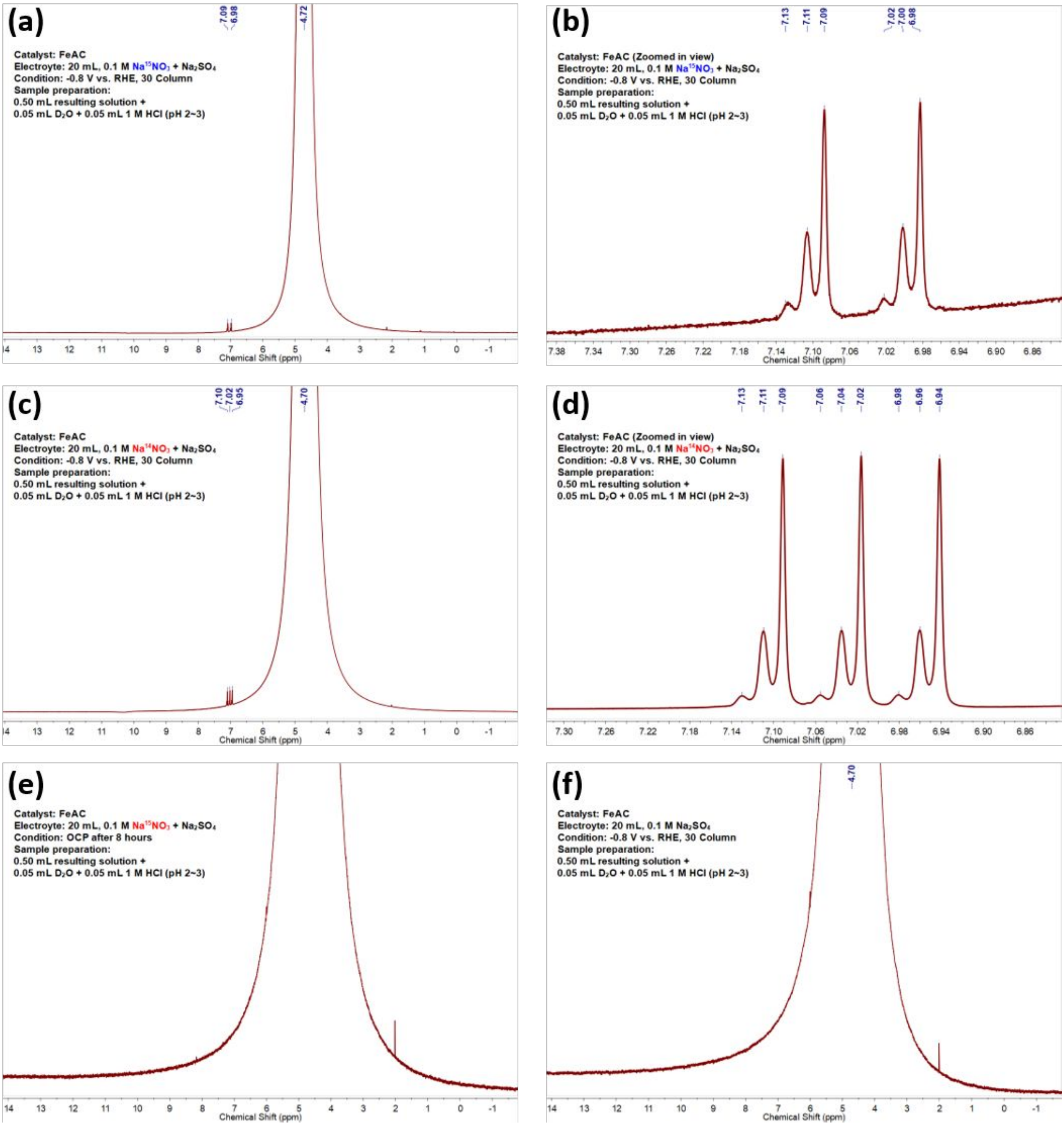

Figure S10. ^1H NMR spectra for the identification of $^{14/15}NH_4^+$ generated by electrochemical nitrate reduction using **FeAC**. (a,c) Full spectra after electrolysis at −0.8 V (vs. RHE) in 0.1 M $Na^{15}NO_3$ + Na_2SO_4 and 0.1 M $Na^{14}NO_3$ + Na_2SO_4, respectively. (b,d) Enlarged views of the $^{15}NH_4^+$ doublet and $^{14}NH_4^+$ triplet regions. Weak adjacent resonances (~0.02 ppm) are attributed to H/D isotopologues of ammonium formed in H_2O/D_2O mixtures during sample preparation. (e) Open-circuit control in $Na^{15}NO_3$ electrolyte for 8 h. (f) Electrolysis in Na_2SO_4 without nitrate. No detectable $^{14/15}NH_4^+$ signals were observed in the control experiments. Prior to NMR analysis, samples were acidified (pH 2–3) with 1 M HCl and mixed with 10% (v/v) D_2O. Spectra were recorded at 700 MHz.

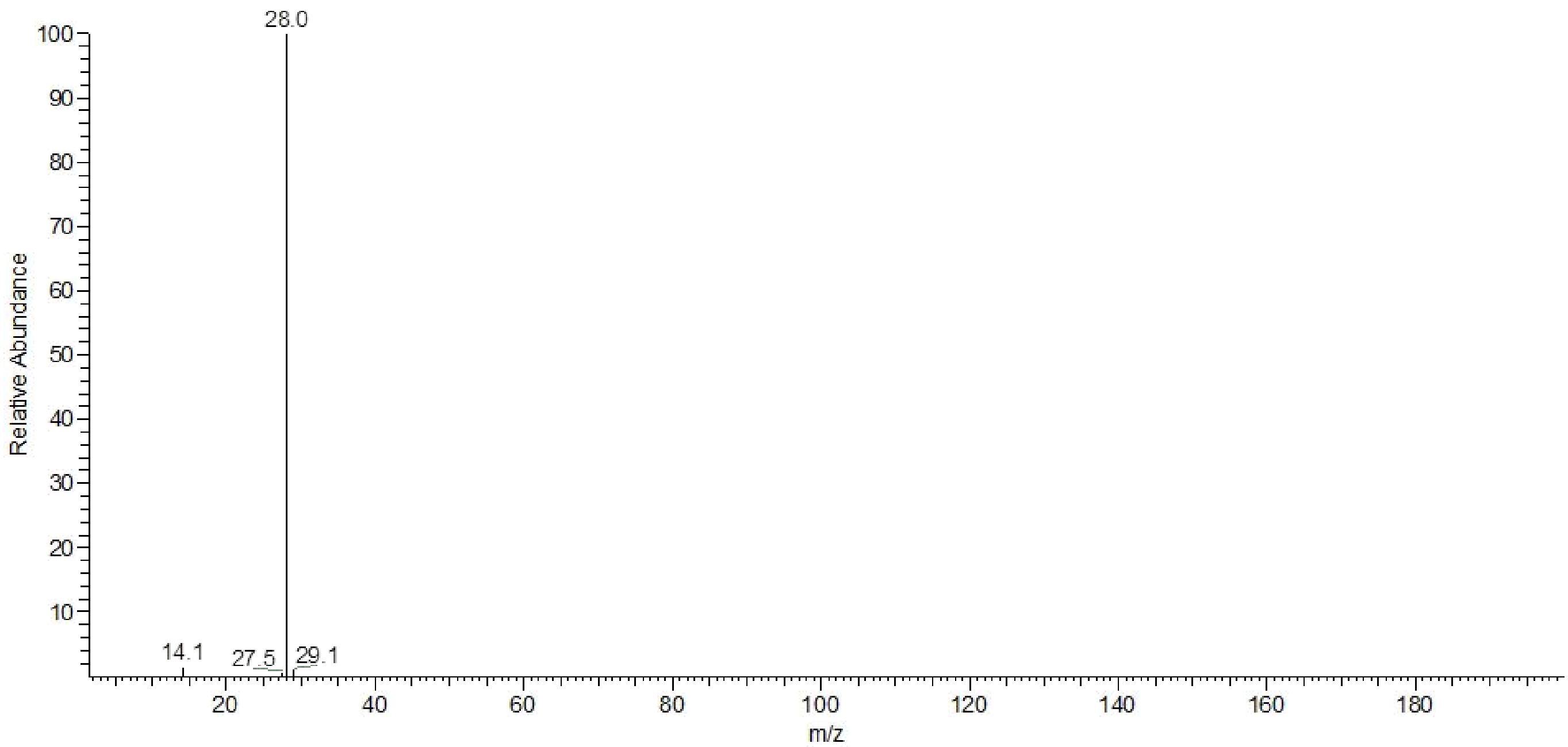

Figure S11. GC-MS of the gaseous products collected after electrolysis of **FeAC** at −0.80 V versus RHE in 0.1 M Na_2SO_4 containing $Na^{15}NO_3$. No detectable signal corresponding to $^{15}N_2$ (m/z = 30) was observed. The signal at m/z = 28 originated from the sampling and instrumental background.

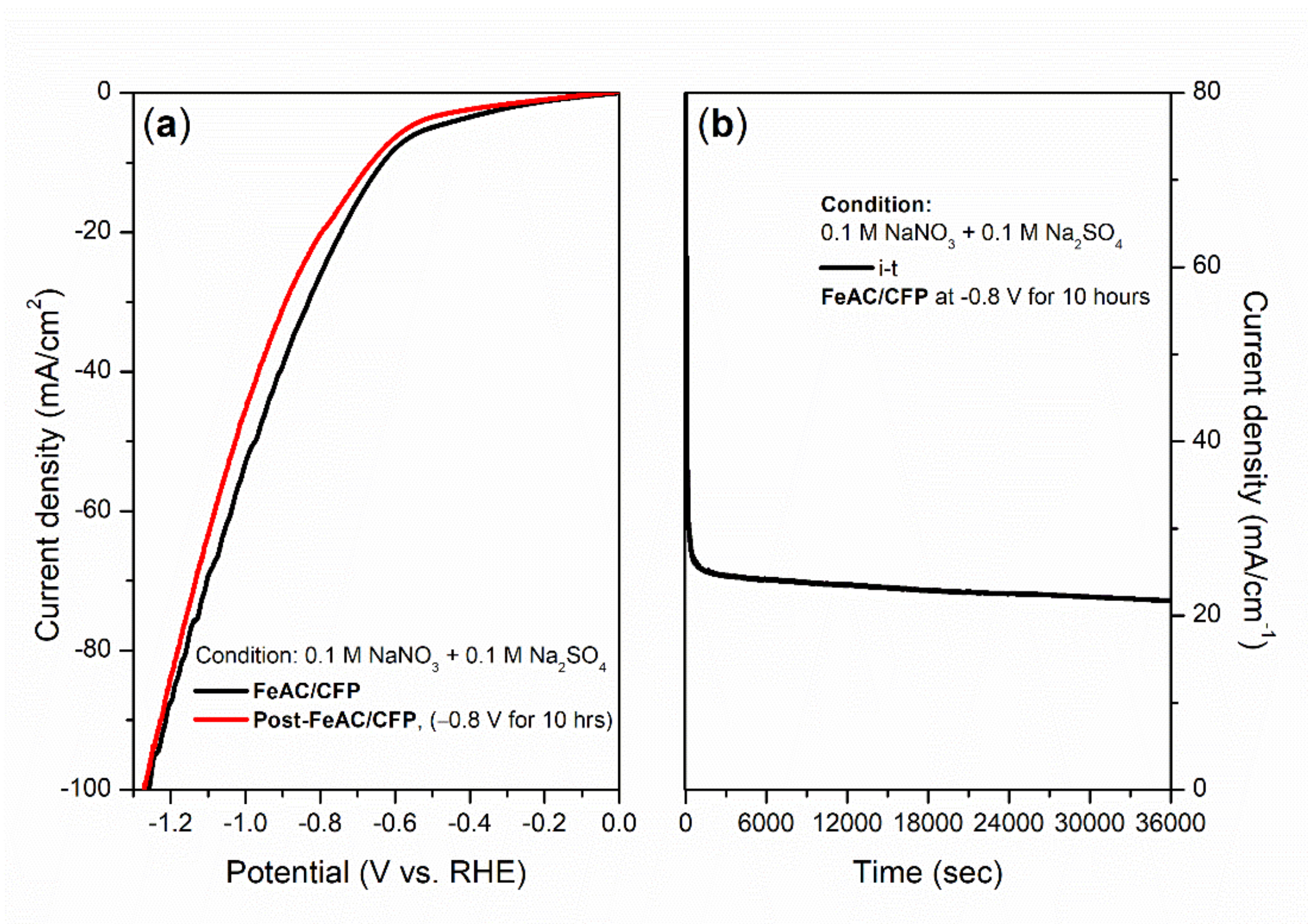

Figure S12. (a) LSV curves of **FeAC/CFP**, and **post-FeAC/CFP** in 0.1 M $NaNO_3$ + 0.1 M Na_2SO_4 electrolyte. (b) longevity test.

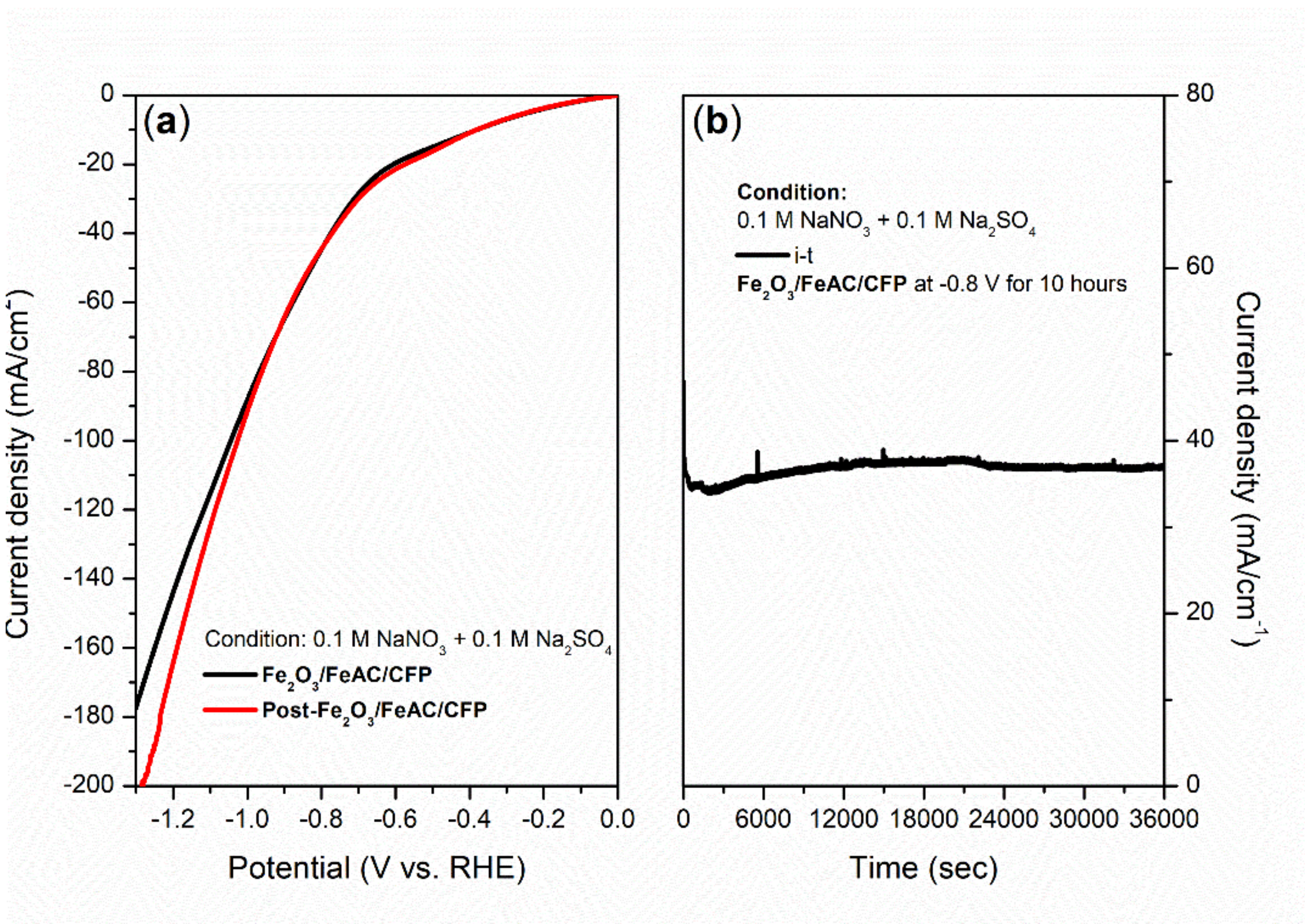

Figure S13. (a) LSV curves of **Fe_2O_3/FeAC/CFP**, and **post-Fe_2O_3/FeAC/CFP** in 0.1 M $NaNO_3$ + 0.1 M Na_2SO_4 electrolyte. (b) longevity test.

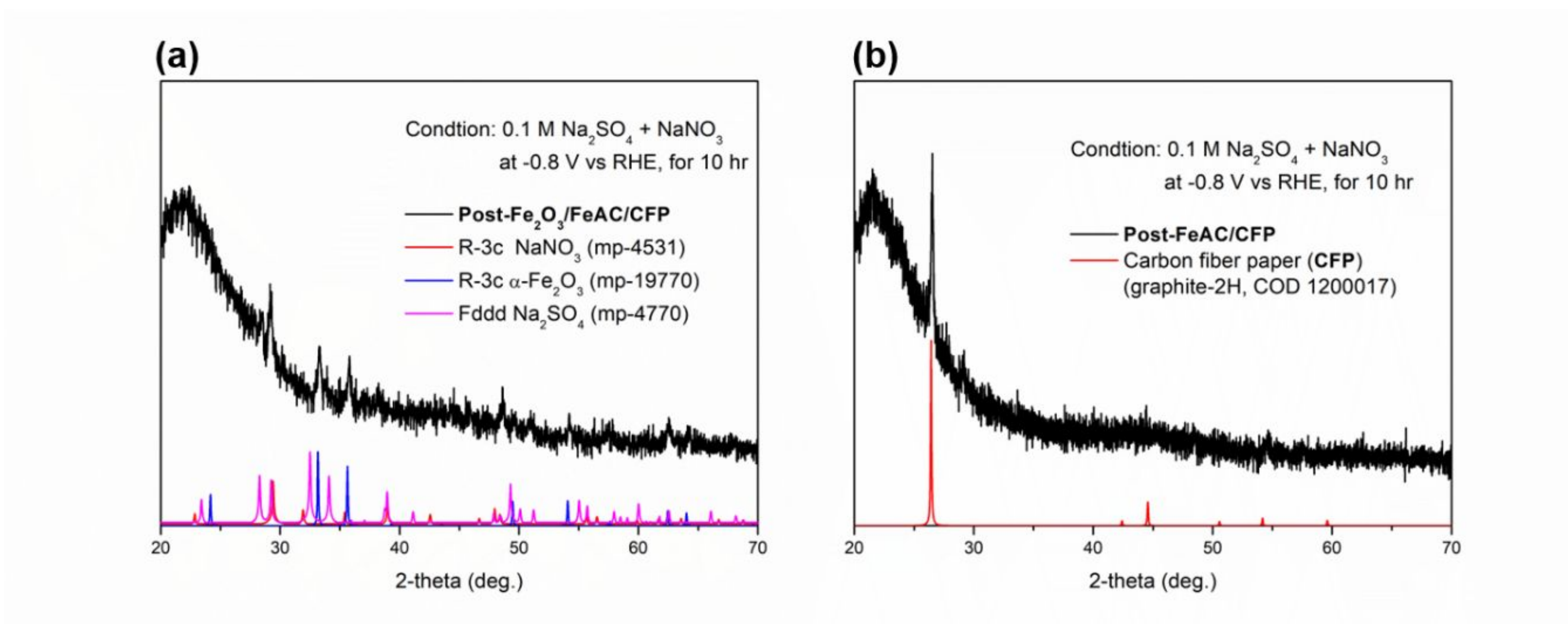

Figure S14. PXRD patterns of (a) **post-Fe_2O_3-FeAC** and (b) **post-FeAC.** The corresponding to reference patterns of residual electrolytes of $NaNO_3$ (R-3c), Na_2SO_4 (Fddd) and the bare carbon fiber paper substrate **(CFP)** of graphite-2H (*$P6_3mc$*).

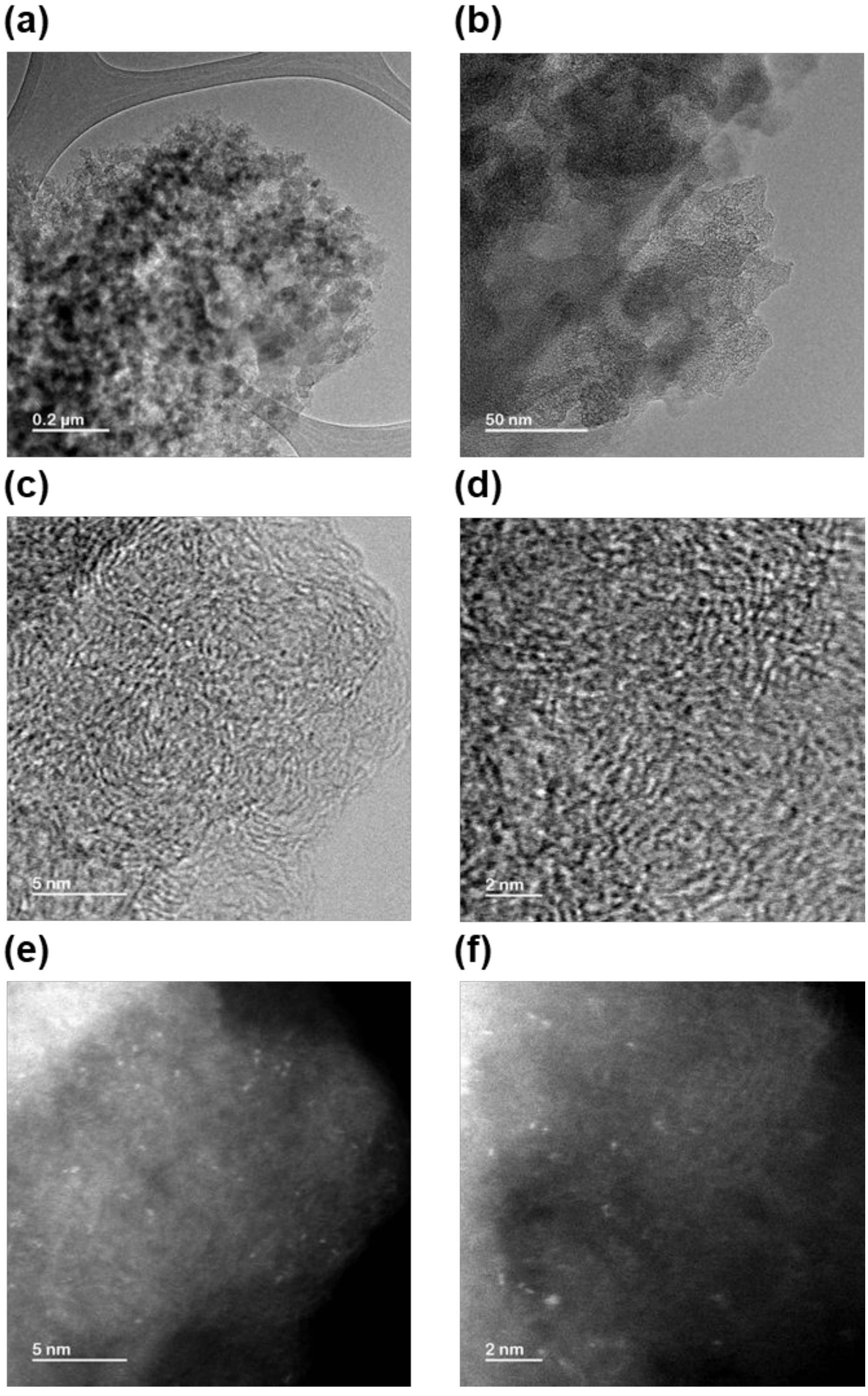

Figure S15. AC-TEM image of **post-FeAC** at (a) low magnification and (b) middle magnification, showing the aggregated carbonaceous morphology. High-magnification bright-field TEM image of selected areas showing the disordered carbon structure without observable Fe nanoparticles. (e, f) High-magnification HAADF-STEM images (from c and d) showing isolated bright Fe features distributed throughout the carbon matrix. The TEM specimen of **post-FeAC** was prepared by dispersing the powder sample in ethanol, followed by drop-casting onto a lacey carbon-coated copper grid and drying under room temperature.

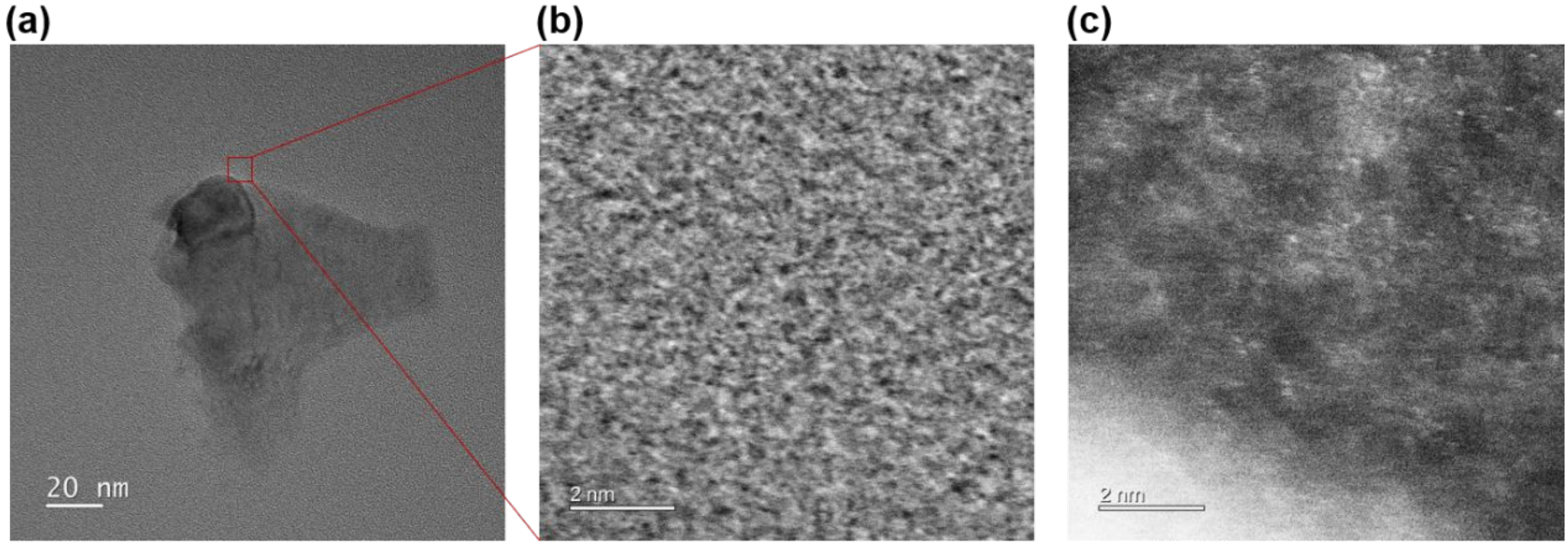

Figure S16. AC-TEM image of **post-Fe_2O_3/FeAC** at (a) low magnification showing the Fe_2O_3 NPs dispersed on carbon-based substrate. (b) High-magnification bright-field TEM image of selected areas showing the disordered carbon structure without observable Fe nanoparticles. (c) High-magnification HAADF-STEM images (from b) showing sparse bright Fe features distributed throughout the carbon matrix. The **post-Fe_2O_3/FeAC** specimen was prepared by focused ion beam (FIB) sectioning to obtain electron-transparent cross-sectional regions for high-resolution TEM analysis.

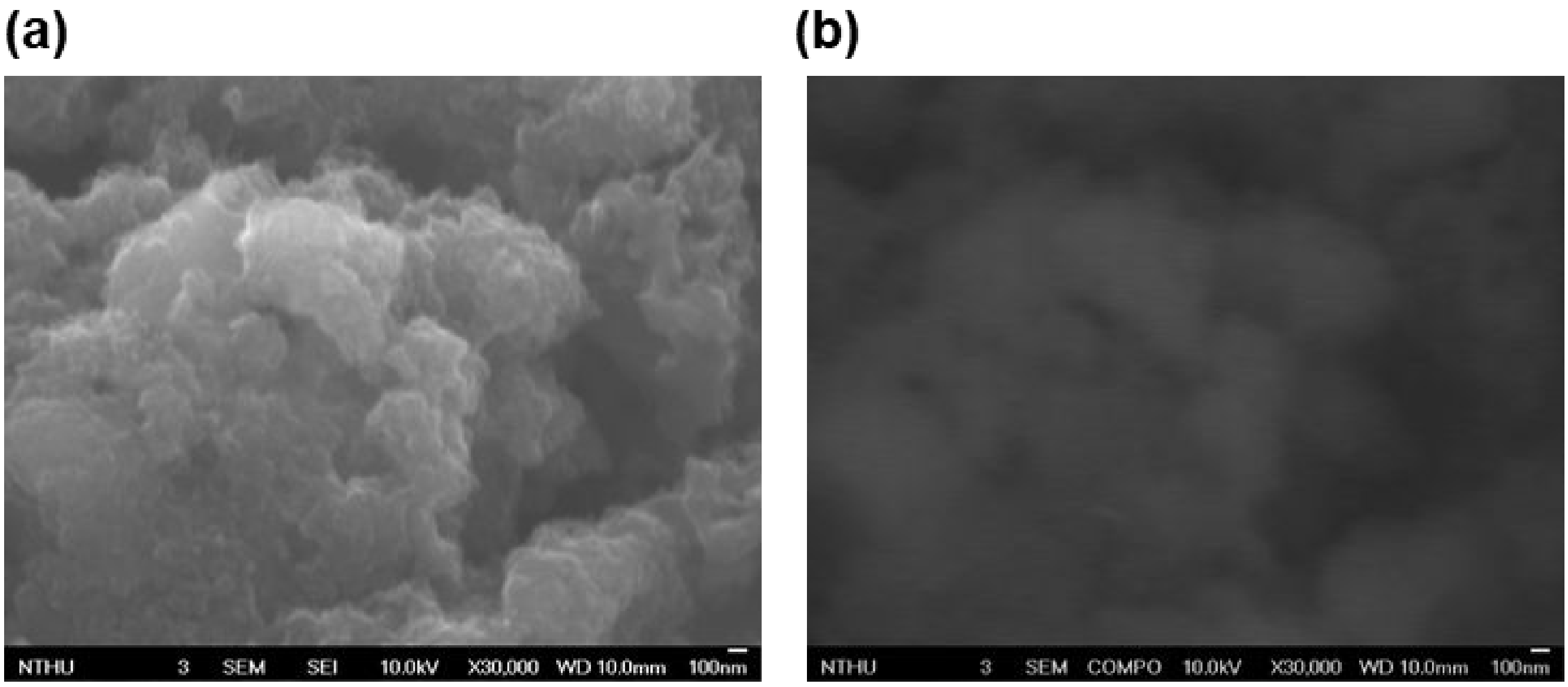

Figure S17. SEM images of the **post-FeAC** acquired in (a) conventional SE mode and (b) BSE mode. The **post-FeAC** catalyst powder was separated from the CFP substrate, dispersed in ethanol by ultrasonication, drop-cast onto a Si wafer, dried, and used for SEM characterization.

(a) (b)

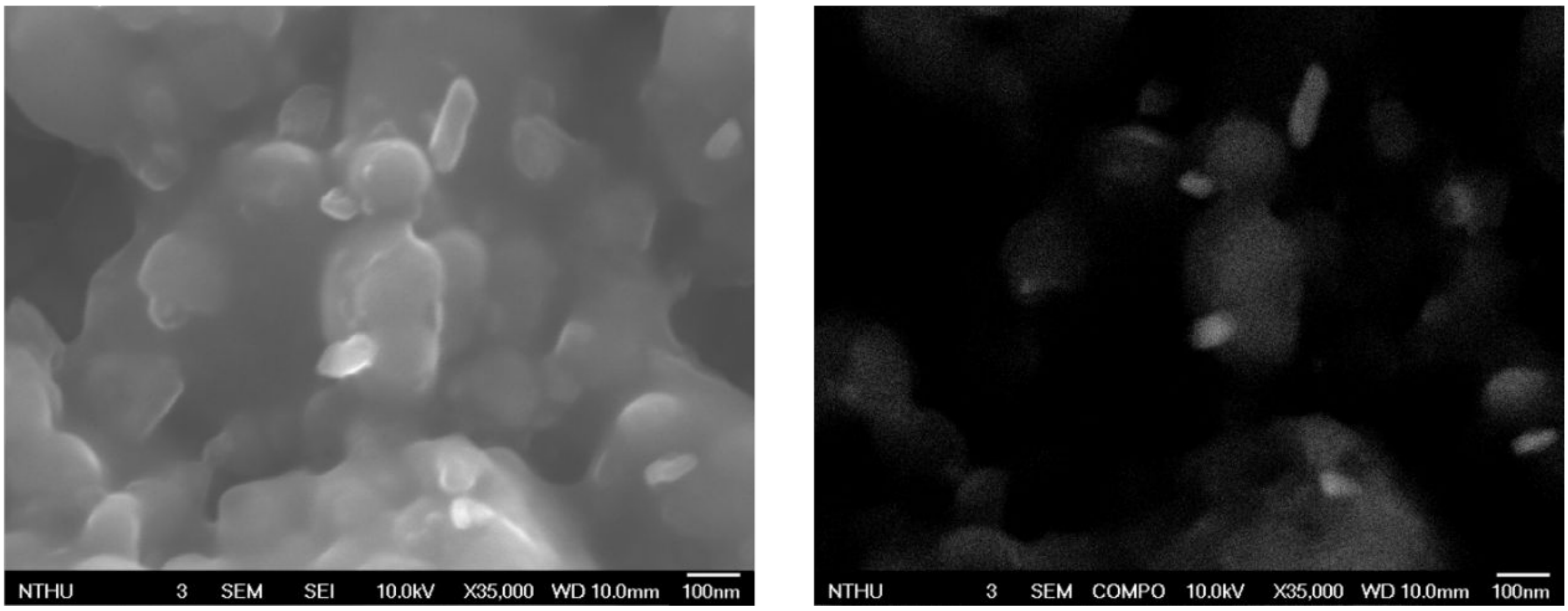

Figure S18. SEM images of the **post-Fe_2O_3/FeAC** acquired in (a) conventional SE mode and (b) BSE mode. The **post-Fe_2O_3/FeAC** catalyst powder was separated from the CFP substrate, dispersed in ethanol by ultrasonication, drop-cast onto a Si wafer, dried, and used for SEM characterization. Iron oxide particle size ranging from 80 nm to 130 nm was found.

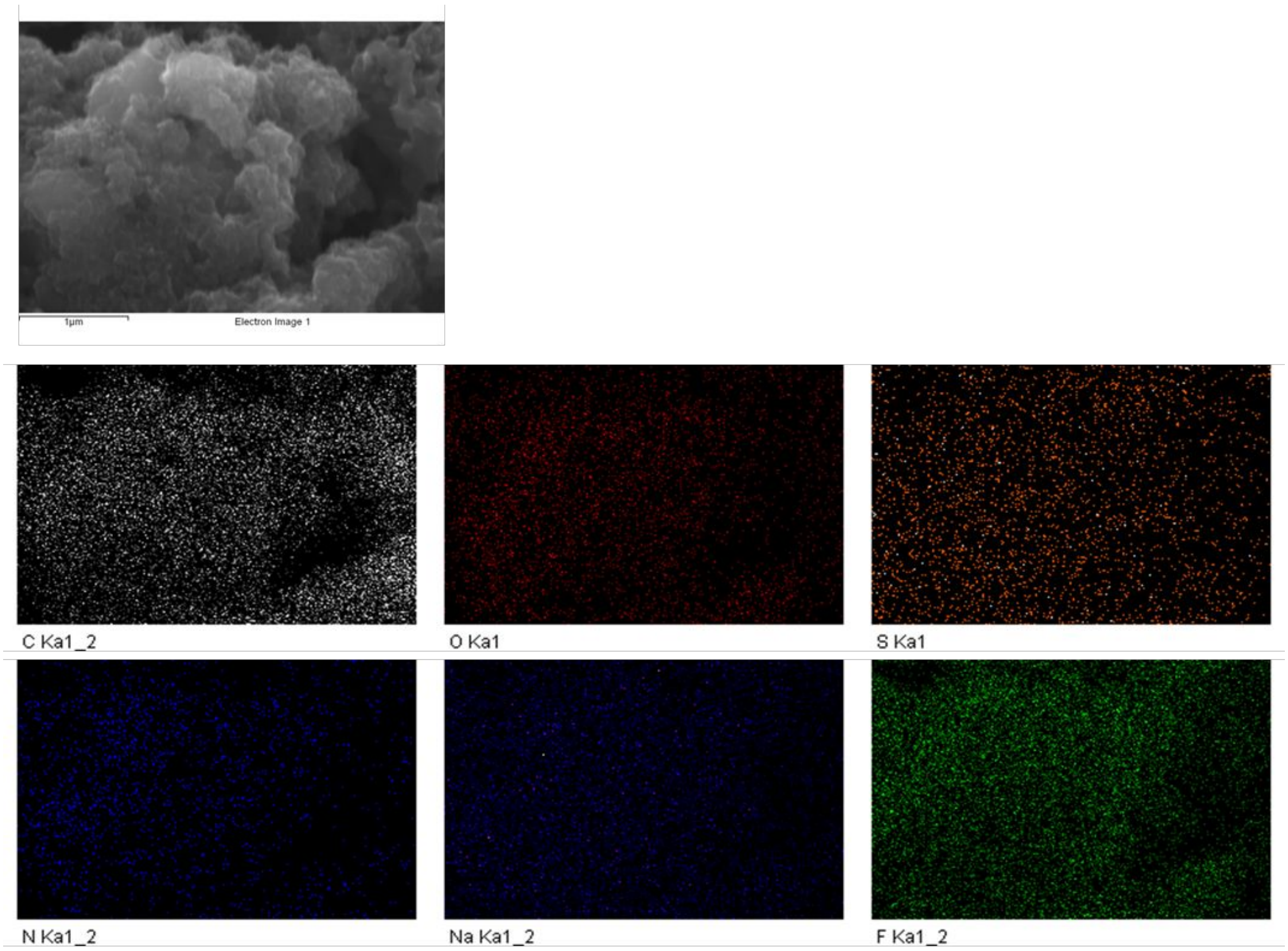

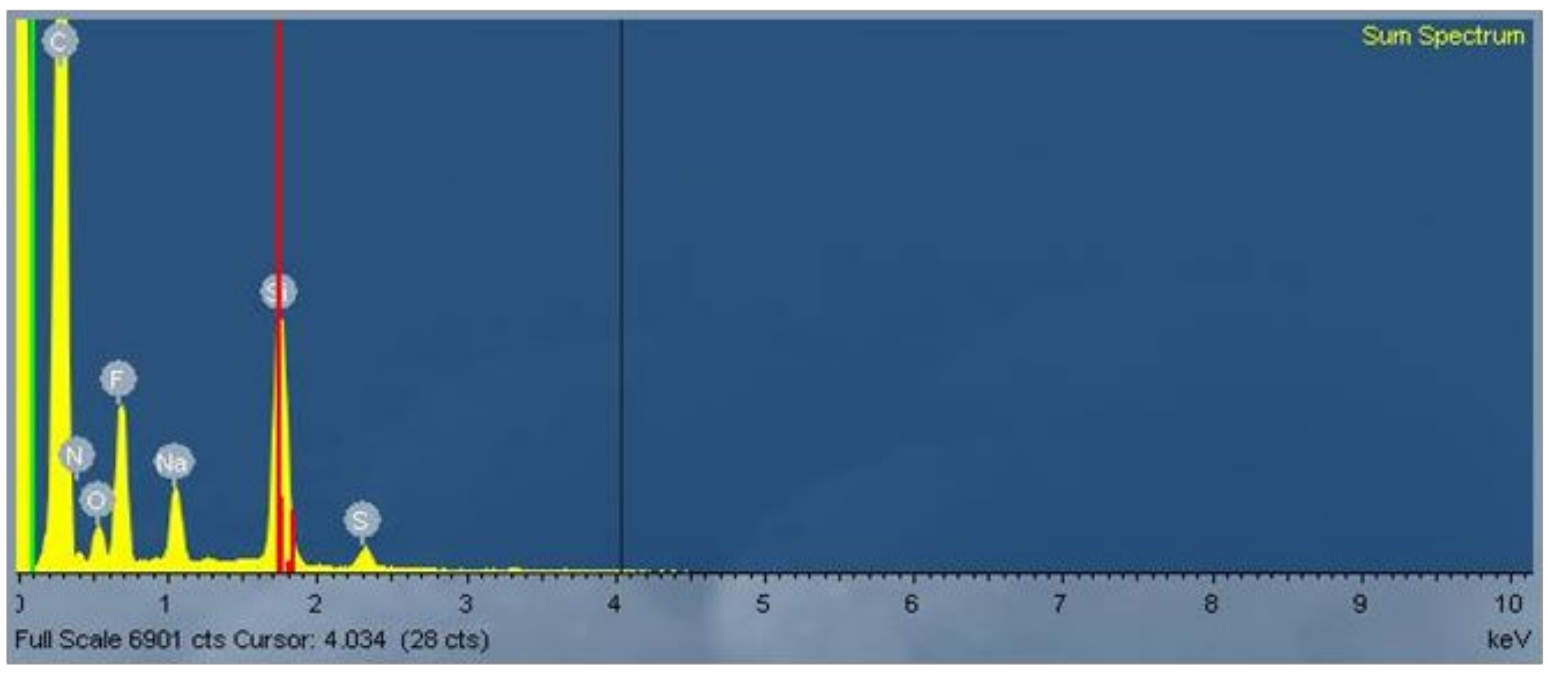

Element	Atomic %	Weight %
C K	75.75	67.18
N K	6.27	6.49
O K	3.66	4.32
F K	11.07	15.52
Na K	1.81	3.08
S K	1.44	3.41

Figure S19. SEM image, corresponding EDX elemental mapping images (C, N, O, F, Na, and S), EDX spectrum, and quantitative elemental composition of the **post-FeAC** catalyst. The **post-FeAC** catalyst powder was separated from the CFP substrate, dispersed in ethanol by ultrasonication, drop-cast onto a Si wafer, dried, and used for SEM-EDX characterization. The presence of F and Na, together with the elevated S content, originates from the Nafion binder and Na_2SO_4 electrolyte.

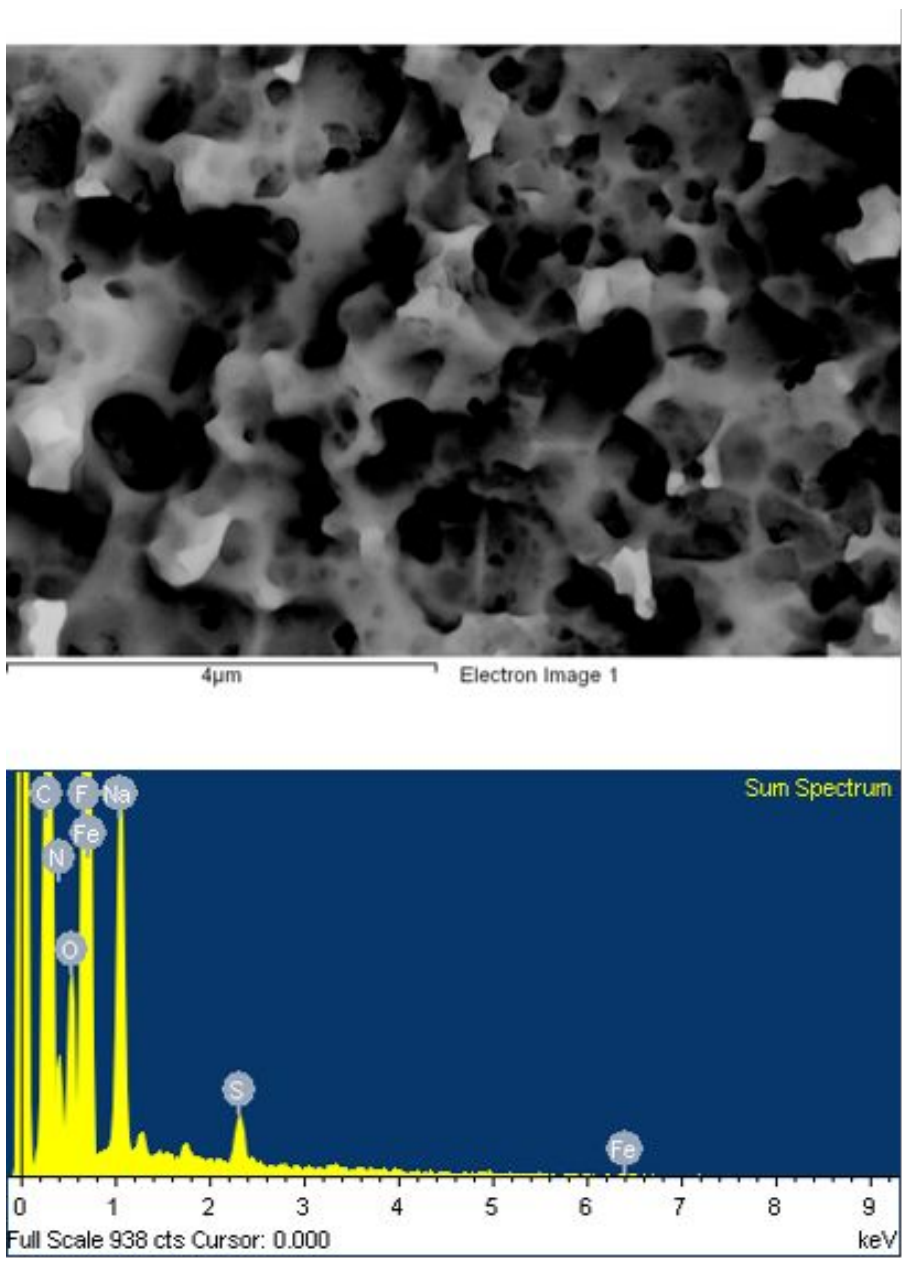

Element	Weight%	Atomic%
C K	40.28	51.61
N K	5.52	6.06
O K	6.11	5.63
F K	38.61	31.27
Na K	6.48	4.34
S K	1.24	0.59
Fe L	1.76	0.50

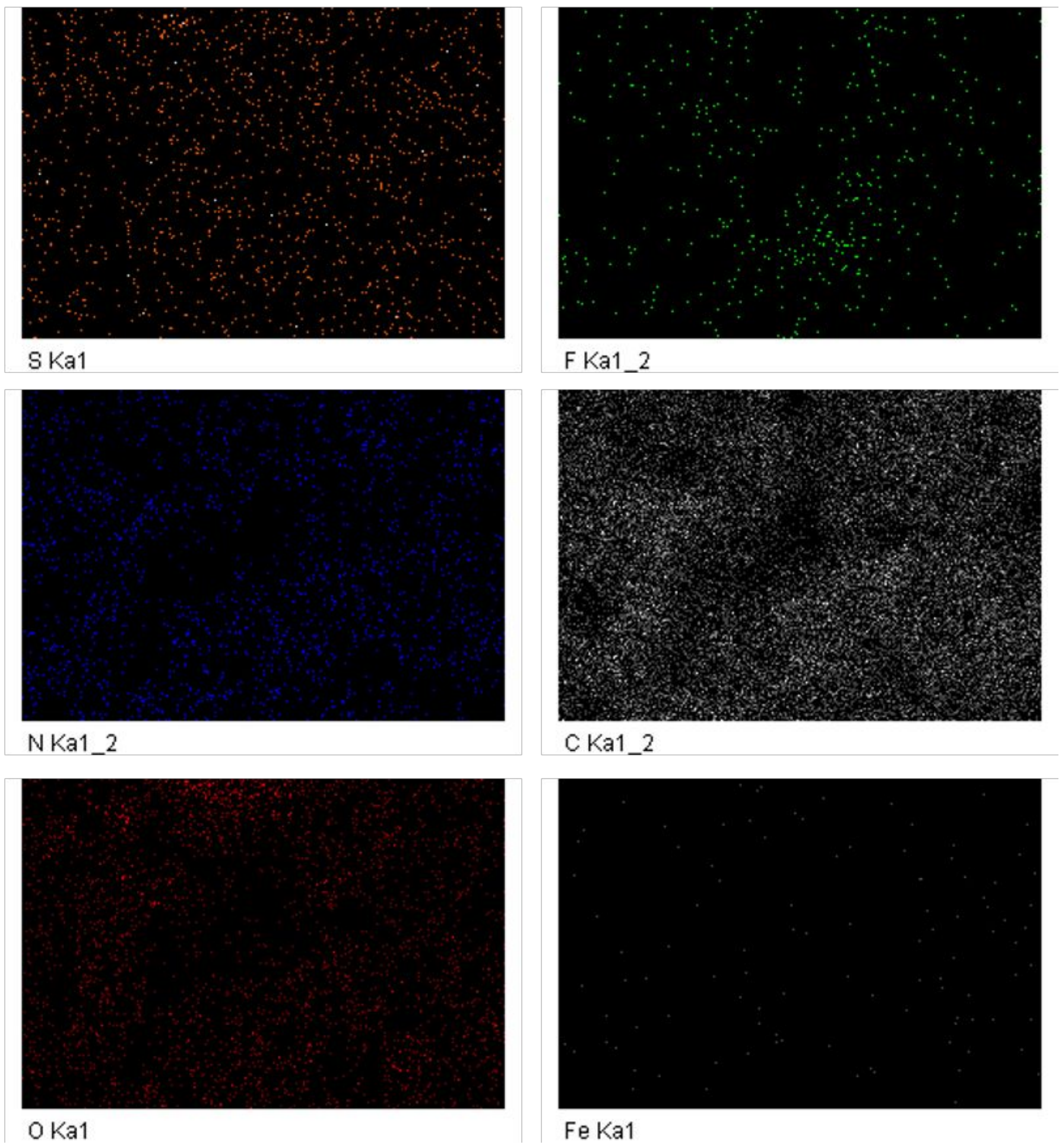

Figure S20. SEM image, corresponding EDX elemental mapping images (C, N, O, F, Na, S, and Fe), EDX spectrum, and quantitative elemental composition of the **post-Fe_2O_3/FeAC** catalyst. The **post-Fe_2O_3/FeAC** catalyst powder was separated from the CFP substrate, dispersed in ethanol by ultrasonication, drop-cast onto a Si wafer, dried, and used for SEM-EDX characterization. The presence of F and Na, together with the elevated S content, originates from the Nafion binder and Na_2SO_4 electrolyte.

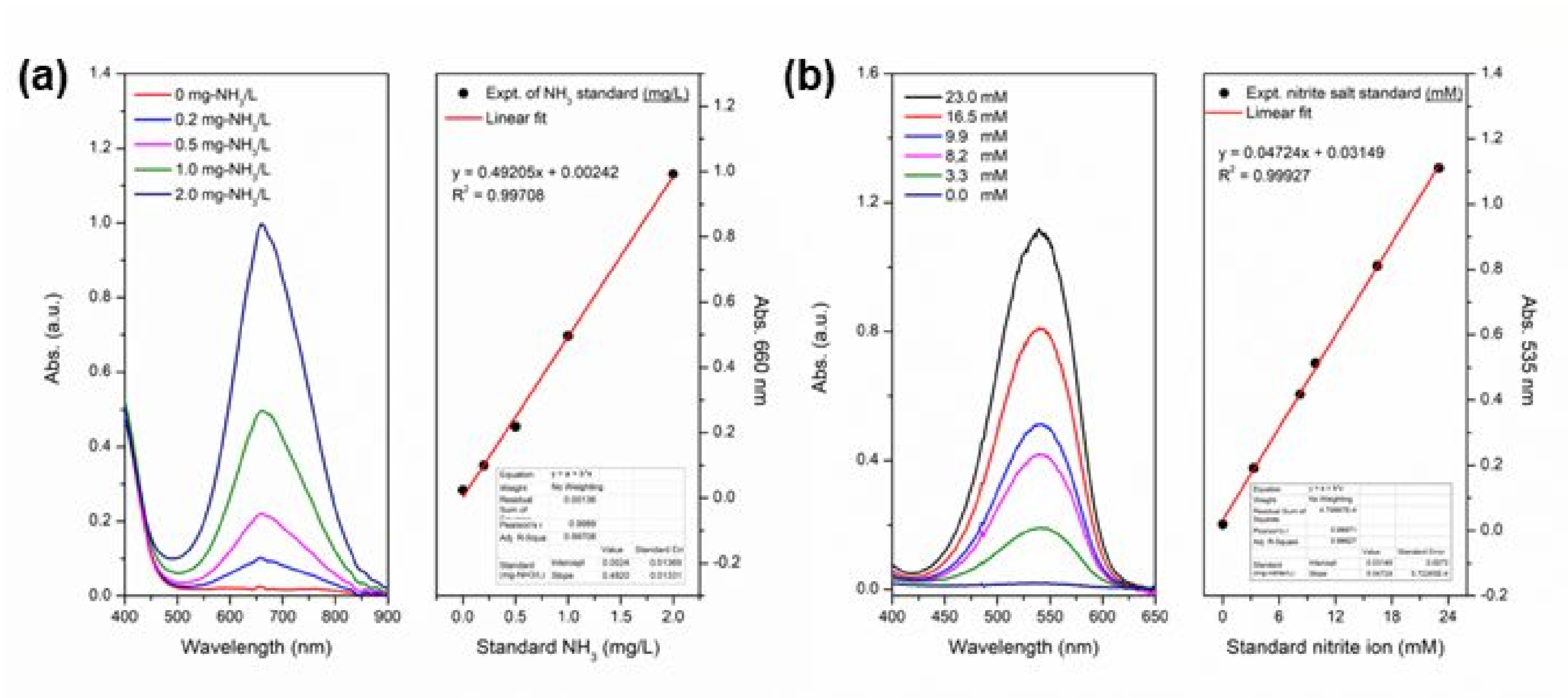

Figure S21. (a) Calibration curve of qualification of liberated NH_3/ NH_4^+ established by indophenol blue method and the different concentrations of NH_4Cl in H_2O and based on UV-Vis absorbance of 630 nm. (b) Calibration curve established qualification of liberated NO_2^- by reactions of Griess reagent and the different concentrations of $NaNO_2$ in H_2O and based on UV-Vis absorbance of 540 nm.

Table S1. Experimental details for as-prepared samples.

	SCB	Fe/PoPDA	Soaking PoPDA in $FeCl_3$ solutions of varying concentrations, followed by isolation
**FeAC**	40 mg	40 mg	200 mg PoPDA + 0.02 mM, 20 mL $FeCl_3$, ultrasonication
**Fe_2O_3/FeAC**	40 mg	40 mg	200 mg PoPDA + 0.10 mM, 20 mL $FeCl_3$, ultrasonication

Table S2. Elemental content of pre-catalysts and post-catalysts analyzed by ICP-MS.

	**Fe (wt%)**
**FeAC**	0.88 ± 0.05
**Fe_2O_3/FeAC**	1.52 ± 0.04
**post-FeAC**	0.79 ± 0.03
**post-Fe_2O_3/FeAC**	1.38± 0.06

Table S3. Comparison of NH_3 yield and FE of different Fe/C-based catalysts for electrochemical NO_3RR.

Electrocatalyst	Electrolyte	FE(NH_3)	Potential (V vs. RHE)	Ref
**FeAC**	0.1 M $NaNO_3$ + 0.1 M Na_2SO_4	79.6	–0.8	This work
**Fe_2O_3/FeAC**	0.1 M $NaNO_3$ + 0.1 M Na_2SO_4	37.8	–0.8	This work
FeOOH/CP	0.1 M $NaNO_3$ + 0.1 M PBS	94.3	–0.4	2
Fe@N_{10}-C	500 ppm $NaNO_3$ + 0.1 M Na_2SO_4	85.2	–0.75	3
Fe SAC	0.5 M KNO_3 + 0.25 M K_2SO_4	75	–0.66	4
Fe_2O_3/Fe–N–C	0.16 M KNO_3 + 1.0 M KOH	> 95	–0.40 to –1.20	5
Fe_1/NC-900	0.5 M KNO_3 + 0.1 M K_2SO_4	86	–0.7	6
Fe/NHCTs	0.1 M $NaNO_3$ + 0.2 M Na_2SO_4	~80	–1.2	7
Fe/NHCTs	0.1 M $NaNO_3$ + 0.1 M HCl	~80	–1.4	7
Fe_{NC}/Fe-Nx	0.5 M $NaNO_3$ + 0.10 M K_2SO_4	93.6	–0.9	8
Fe–N–C/γ-Fe_2O_3	0.1 M KNO_3 + 0.1 M KOH	90.6	–0.7	9
Fe_{NP}/Fe_{SA}-N-C	0.1 M KNO_3 + 0.5 M K_2SO_4	95.5	–0.85	10
Fe_1/NCB_d@CNT-FEM	50 mM Na_2SO_4 or 50 mM Na_2SO_4	>99	–1.1 vs. Ag/AgCl	11
γ-Fe_2O_3/BCN	0.1 M $NaNO_3$ + 0.1 M PBS	95	–0.59	12
Fe_2O_3/CC	0.1 M $NaNO_3$ + 0.5 M Na_2SO_4	84.87	–0.9	13

Reference

(1) Powder X-ray diffraction database was downloaded from Material Project and Crystallography Open Database (https://next-gen.materialsproject.org/ and https://www.crystallography.net/cod/index.php ; Fe: mp-13; Fe_3O_4: mp-19306; Fe_2O_3: mp-19770; FeO: mp-18905; graphite-2H: COD 1200017).

(2) Liu, Q.; Liu, Q.; Xie, L.; Ji, Y.; Li, T.; Zhang, B.; Li, N.; Tang, B.; Liu, Y.; Gao, S.; Luo, Y.; Yu, L.; Kong, Q.; Sun, X., High-Performance Electrochemical Nitrate Reduction to Ammonia under Ambient Conditions Using a FeOOH Nanorod Catalyst. *ACS Appl. Mater. Interfaces.* **2022**, 14 (15), 17312-17318.

(3) Shuo Zhang; Miao Lia; Jiacheng Li; Qinan Song;Liu,X., N-doped carbon-iron heterointerfaces for boosted electrocatalytic active and selective ammonia production. *Proc. Natl. Acad. Sci. U.S.A.*. **2023**, e2207080119.

(4) Wu, Z.-Y.; Karamad, M.; Yong, X.; Huang, Q.; Cullen, D. A.; Zhu, P.; Xia, C.; Xiao,Q.; Shakouri, M.; Chen, F.-Y.; Kim, J. Y.; Xia, Y.; Heck, K.; Hu, Y.; Wong, M. S.; Li, Q.; Gates, I.; Siahrostami, S.;Wang, H., Electrochemical ammonia synthesis via nitrate reduction on Fe single atom catalyst. *Nat. Commun*. **2021**, 12 (1), 2870.

(5) E. Murphy, B. Sun, M. Rüscher, Y. Liu, W. Zang, S. Guo, Y.-H. Chen, U. Hejral, Y. Huang, A. Ly, I. V. Zenyuk, X. Pan, J. Timoshenko, B. R. Cuenya, E. D. Spoerke, P. Atanassov, Synergizing Fe_2O_3 Nanoparticles on Single Atom Fe-N-C for Nitrate Reduction to Ammonia at Industrial Current Densities. *Adv. Mater.* **2024**, 36, 2401133.

(6) Liu, L.; Xiao, T.; Fu, H.; Chen, Z.; Qu, X.; Zheng, S. Construction and identification of highly active single-atom Fe1-NC catalytic site for electrocatalytic nitrate reduction. *Appl. Catal. B: Environ.* **2023**, *323*, 122181.

(7) Zhang, H.; Liu, Y.; Gao, S.; Wang, H.; Bai, R.; Zhao, Y.; Zhou, Y.; Hu, G.; Zhao, X. Atomically dispersed iron & iron clusters synergistically accelerate electrocatalytic ammonia synthesis. *Chem. Eng. J.* **2025**, *504*, 158785.

(8) Song, B.; Xiang, D.; Chen, C.; Xu, J.; Ma, Y.; Yang, L.; Lee, M.-H.; DengkeZhao; Han, K.; Wang, N. Enhancing nitrate reduction reaction electroactivity of Fe single atom catalyst by modification of Fe nanoclusters and further application in self-powered ammonia synthesis from air. *Chem. Eng. J.* **2025**, *521*, 166374.

(9) Ma, C.; Zhong, X.; Guan, Y.; Wu, F.; Liu, J.; Zuo, M.; Liu, J. Tunable-crystallinity N-doped carbon supports synergizing with Fe active sites for efficient nitrate electroreduction. *Appl. Catal. B: Environ.* **2026**, *384*, 126223.

(10) Wang, L.; Guo, P.; Han, Y.; Han, C.; Sun, H.; Huang, R.; Liu, X.; Huang, M.; Mao, Z.; Yan, X.; Du, A.; Wang, X. Coupling single-atomic iron sites with iron nanoparticles for tandem-enhanced ammonia electrosynthesis from nitrate. *Chem Catalysis* **2024**, *4* (4), 100936.

(11) Wang, X.; Winter, L. R.; Wu, X.; Fan, Y.; Zhao, Y.; Kim, J.-H.; Elimelech, M. Intensified atomic

utilization efficiency of single-atom catalysts for nitrate conversion via electrified nanoporous membrane. *Sci. Adv.* **2025**, *11* (28), eads6943.

(12) Mariya Varghese, S.; Gopinathan, A. V.; Rathnakumaran, S.; Sasikumar, S.; Kunnikuruvan, S.; Gangavarapu, R. R. Electrochemical Nitrate Reduction to Ammonia using γ-Fe2O3/BCN in a Neutral Medium. *ACS Catal.* **2025**, *15* (8), 5982-5992.

(13) Li, T.; Tang, C.; Guo, H.; Yang, J.; Zhang, F.; Yang, G.; Zhou, Y. Understanding first electron transfer kinetic process of electrochemical nitrate reduction to ammonia on Fe2O3 nanorods array. *Chem. Eng. J.* **2024**, *485*, 149560.
